# *Smilax glabra* Roxb. Flavonoids Attenuate D-Galactose-Induced Cardiac Alterations and Are Associated with Modulation of the PGC-1α/NRF1/TFAM Axis and PINK1/Parkin Signaling Pathway in an AMPK-Associated Manner

**DOI:** 10.3390/ijms27219969

**Published:** 2026-11-06

**Authors:** Danting Fu, Jiajun Shi, Qinqin Yang, Chunwei Zheng, Yu Chen

**Affiliations:** Department of Experimental Animals, Zhejiang Academy of Traditional Chinese Medicine, Hangzhou 310007, China; fdt1018@163.com (D.F.); xsrjsjj1992@126.com (J.S.); qqy1029@126.com (Q.Y.); zhengcw1972@163.com (C.Z.)

**Keywords:** *Smilax glabra* Roxb., D-galactose, cardiac alteration, AMPK, PGC-1α/NRF1/TFAM axis, PINK1/Parkin signaling pathway

## Abstract

*Smilax glabra* Roxb. flavonoids (SGF) are the rhizome extracts of *Smilax glabra* Roxb. and possesses pharmacological activity, prior studies have demonstrated SGF’s cardioprotective potential, specifically its capacity to inhibit pathological cardiac hypertrophy. Building upon this evidence, this study investigated whether SGF exerts protective effects against D-galactose-induced cardiac alterations and elucidated its underlying molecular mechanisms. D-galactose was employed to induce aging-like changes in C57BL/6J mice and H9c2 cell. The cardioprotective effects of SGF against D-galactose-induced myocardial alterations were investigated, with particular emphasis on mitochondrial ultrastructural integrity, expression of key factors in the PGC-1α/NRF1/TFAM axis and PINK1/Parkin signaling pathways, in relation to AMPK signaling. Additionally, in vitro, the AMPK inhibitor Compound C and the AMPK agonist AICAR were used to examine whether the cardioprotective effect of SGF was associated with AMPK. In vivo, SGF administration significantly attenuated D-galactose-induced cardiac hypertrophy, ameliorated myocardial fibrosis and ROS overproduction, preserved mitochondrial ultrastructure, enhanced AMPK phosphorylation (increased p-AMPKα/AMPK ratio), and upregulated the expression of key proteins involved in the PGC-1α/NRF1/TFAM axis and PINK1/Parkin signaling pathways. In vitro, SGF effectively suppressed D-galactose-induced senescence-like changes in H9c2 cells, these beneficial effects were abolished upon co-treatment with Compound C, and the action of SGF was similar to that of AICAR. These results suggested that SGF attenuated D-galactose-induced cardiac hypertrophy and preserves mitochondrial structure. These effects were accompanied by increased AMPK phosphorylation and increased expression of proteins related to PGC-1α/NRF1/TFAM axis and PINK1/Parkin signaling pathway. These changes were consistent with an AMPK-associated pattern and may contribute to the cardioprotective effects of SGF via effects on mitochondrial function.

## 1. Introduction

Aging is a common and inevitable physiological process, accompanied by the degradation of substances, morphology, and function in cells, tissues, and organs, as well as a reduction in adaptability and resistance. With technological advancements and improvements in quality of life in modern society, people’s lifespans have been extended, making the issue of aging a global focal point in the field of life sciences. Among these concerns, cardiovascular diseases have consistently been the leading cause of death among the elderly, posing significant challenges to healthy and resulting in substantial consumption of medical resources [1]. Cardiac aging has also become an independent risk factor for cardiovascular diseases, if left unaddressed, it will ultimately progress to heart failure and result in death [2]. Although aging cannot be halted, slowing down its pace is entirely achievable. Therefore, exploring effective preventive and therapeutic approaches to mitigate cardiac aging is of paramount importance for reducing the prevalence of cardiovascular diseases and alleviating the burden on social healthcare systems.

In myocardial cells, mitochondria are the most abundant among all organ tissues, accounting for approximately one-third of the volume of myocardial cells, and providing a substantial amount of energy for the heart. Therefore, changes in mitochondrial structure and function are also considered significant factors influencing the development of cardiovascular diseases [3,4,5]. Mitochondrial biogenesis and mitophagy are two key mechanisms within mitochondrial quality control [6]. Mitochondrial biogenesis refers to the tightly regulated cellular process of synthesizing new mitochondria to replace damaged or senescent organelles, thereby maintaining mitochondrial quantity, structural integrity, and functional capacity; the PGC-1α/NRF1/TFAM axis is a key pathway regulating mitochondrial biogenesis, in which NRF-1/TFAM are key regulatory factors of mitochondrial biogenesis [7]. Mitophagy is an organelle-specific autophagic process that maintains the balance of mitochondrial quantity and quality by selectively eliminating excess or damaged mitochondria, and the PINK1/Parkin signaling pathway is the most classic pathway mediating mitophagy [8]. The 5′ AMP-activated protein kinase (AMPK) is a natural sensor of cellular metabolism and energy homeostasis. A large number of studies have confirmed that AMPK is widely expressed in the cardiovascular system and participates in the pathogenesis of various cardiovascular diseases [9], including pulmonary hypertension [10], atherosclerosis (AS) [11], ischemia–reperfusion injury [12], and cardiac hypertrophy [13]. It is worth noting that AMPK is closely related to mitochondrial biology, and has close molecular associations with the PGC-1α/NRF1/TFAM axis and the PINK1/Parkin signaling pathway. Studies have found that in a high-fat-induced HFpEF mouse model, AMPK phosphorylation can activate the PGC-1α/NRF1/TFAM axis to ameliorate mitochondrial biogenesis impairment, alleviate mitochondrial functional damage, and improve cardiac function in mice [14]. In ISO-induced H9c2 cells, AMPK activation rescued the PGC-1α/NRF1/TFAM axis and alleviated mitochondrial dysfunction and oxidative stress [15]. In addition, AMPK activation also reduced oxidative stress and promoted mitophagy in the myocardium of doxorubicin-induced cardiac aging mice by mediating the PINK1/Parkin pathway, thereby alleviating myocardial aging [16]. These findings suggest that, in the heart, AMPK is essential for maintaining mitochondrial structure and function. Studies have shown that with age, mitochondria in cardiomyocytes undergo structural and functional changes, such as fragmentation [17], swelling [18], reduced endocardial phospholipid content [19], altered membrane fluidity [20], and decreased oxidative phosphorylation capacity [21]. The abnormal accumulation of these damaged mitochondria will affect the overall mitochondrial dysfunction, further lead to myocardial damage, and contribute to the phenotypic aging of the heart [22,23]. Through some intervention methods, including exercise [24], caloric restriction [25], and anti-aging drugs (resveratrol [26], metformin [27], rapamycin [28], berberine [29], etc.), the heart function of model animals can be improved and lifespan extended, the molecular mechanisms of these interventions typically involve the PGC-1α/NRF1/TFAM axis and the PINK1/Parkin signaling pathway, thereby affecting mitochondrial biogenesis or mitophagy. In addition, studies have found that aging can significantly affect AMPK activity in the heart, negatively impact cellular energy balance, and disrupt mitochondrial homeostasis to aggravate cardiac aging [30]. Therefore, therapeutically, this also suggests that regulating mitochondrial biogenesis and mitophagy through AMPK may be a promising strategy to prevent cardiovascular diseases caused by aging.

Chinese herbal medicine possesses a profound theoretical foundation and cultural background, it holds immense potential for combating aging [31]. For example, Ginkgolide B (GB), derived from Ginkgo biloba, and total ginsenoside (TG), a mixture of the primary active ginsenosides from Panax ginseng, have been proven to extend the lifespan of model organisms [32,33]. *Smilax glabra* Roxb. belongs to the *Smilacaceae* family. It is known as “Tu-fu-ling” in China, and is commonly used as both food and medicine. Modern pharmacology indicates that the rhizome extracts of *Smilax glabra* Roxb. possess various pharmacological properties, including anti-infective, immunomodulatory, antioxidant, and hepatoprotective effects [34]. Our previous studies demonstrated that the *Smilax glabra* Roxb. flavonoids (SGF) alleviated cardiac hypertrophy by inhibiting the Raf/MEK/ERK pathway [35], and suppressed cardiac inflammation through the NLRP3/Caspase-1/IL-18 inflammasome pathway [36]. Moreover, relevant studies indicated that SGF modulated cellular energy homeostasis via the AMPK. Specifically, SGF enhanced AMPK phosphorylation in mouse 3T3-L1 adipocytes and stimulated mitochondrial biogenesis [37]. Concurrently, SGF exerts anti-proliferative effects in vitro and in vivo in obesity-related colorectal cancer by inhibiting AMPK activation, thereby driving ferroptosis [38]. These findings position AMPK as a pivotal molecular target underlying SGF’s pharmacological actions—and potentially mediating its anti-cardiac hypertrophic effects. Although preliminary data suggest cardioprotective properties of SGF, the involvement of AMPK and its linkage to mitochondrial in this context remain incompletely characterized. Therefore, this study employed both in vivo and in vitro experiments to evaluate the anti-cardiac hypertrophy effect of SGF on D-galactose-induced cardiac alterations, investigating the potential mechanism underlying SGF-mediated prevention of cardiac hypertrophy by targeting AMPK signaling, with specific emphasis on its regulatory effects on the mitochondria-related PGC-1α/NRF1/TFAM axis and PINK1/Parkin signaling pathway.

## 2. Results

### 2.1. Changes of Echocardiographic Parameters in Mice at Different Periods of SGF Treatment

As shown in Table 1 and Table 2, cardiac echocardiography was conducted for all mouse groups. After a 4-week treatment period of SGF, the D-galactose group exhibited mild cardiac hypertrophy, with significant thickening of the IVSd, IVSs, LVPWs, and LVPWd. Concurrently, FS and EF demonstrated compensatory elevation. Notably, following 4 weeks of SGF treatment, IVSd, IVSs, LVPWs, and LVPWd were significantly decreased in the SGF-L group and SGF-H group relative to the D-galactose group. After a 8-week treatment period of SGF, cardiac hypertrophy became more pronounced in D-galactose group, while the anti-hypertrophic effect of SGF treatment was more significant, manifested as significantly decreased IVSd, IVSs, LVPWs, and LVPWd, significantly increased LVIDd, LVIDs, LVEDV, and LVESV, and reduced EF and FS. This indicated that SGF had the potential to inhibit cardiac hypertrophy caused by D-galactose.

### 2.2. Effects of SGF on Aging Markers and Histopathological Alterations in D-Galactose Mice

As shown in Figure 1, following an 8-week treatment of SGF, all mice were humanely euthanized under anesthesia, and hearts were harvested for analysis. Compared with the control group, myocardial tissue from the D-galactose group exhibited marked structural disorganization, characterized by loss of cellular alignment, indistinct intercellular boundaries, cardiomyocyte enlargement, and deposition of collagen fibrosis. In addition, the cardiac index (heart weight/body weight ratio) was significantly elevated, mRNA levels of hypertrophy-associated genes (ANP, BNP, and β-MHC) and the aging marker proteins (p21, p16) were significantly upregulated in the myocardial tissue. After treatment with SGF, the morphology of the mouse cardiomyocytes improved, with a reduction in cell area and a decrease in collagen fibrosis deposition. Concurrently, the expression levels of hypertrophy-associated genes (ANP, BNP, and β-MHC) were downregulated, and protein expression of the aging marker proteins (p21, p16) was markedly decreased. Collectively, these findings demonstrated that SGF alleviated D-galactose-induced cardiac hypertrophy in mice and reduced the overexpression of aging marker proteins.

### 2.3. Effects of SGF on ROS Expression in the Myocardium of D-Galactose Mice

As shown in Figure 2, D-galactose stimulation disrupted redox homeostasis in mouse cardiomyocytes, leading to a significant increase in ROS levels. After treatment with SGF, the excessive production of ROS in the myocardial tissue was effectively reduced.

### 2.4. Effect of SGF on Myocardial Injury Markers in Serum of D-Galactose Mice

As shown in Figure 3, serum levels of the heart failure marker NT-proBNP were significantly elevated in the D-galactose group relative to the control group. Concurrently, the myocardial injury markers cTnT and CK-MB, as well as the inflammatory marker CRP, were also markedly increased. In contrast, SGF treatment effectively attenuated these pathological alterations, resulting in significant reductions in all aforementioned markers.

### 2.5. Effects of SGF on the Expression of Key Factors in the AMPK and PGC-1α/NRF1/TFAM Axis in D-Galactose Mice

After treatment with D-galactose, we found through transmission electron microscopy that the overall structure of mouse myocardial mitochondria showed a tendency of swelling in the D-galactose group, mitochondrial cristae became loose and disordered, the electron density of the matrix decreased; meanwhile, AMPK phosphorylation in myocardial tissue was inhibited, with a reduced p-AMPKα/AMPK ratio, as shown in Figure 4. The protein expression of key factors in the PGC-1α/NRF1/TFAM axis—a core pathway of mitochondrial biogenesis—namely PGC-1α, NRF1, and TFAM, was significantly downregulated. In contrast, SGF treatment ameliorated these ultrastructural mitochondrial impairments, restored the p-AMPKα/AMPK ratio, and upregulated the expression of PGC-1α, NRF1, and TFAM. Collectively, these results suggest that SGF may act through AMPK to influence the PGC-1α/NRF1/TFAM axis, thereby affecting mitochondria.

### 2.6. Effect of SGF on Key Factors of AMPK/ULK1-Mediated PINK1/Parkin Signaling Pathway in D-Galactose Mice

Extant literature indicates that the AMPK/ULK1 pathway plays a regulatory role in PINK1/Parkin-dependent mitophagy [39]. Therefore, we investigated the effects of SGF on ULK1 protein, the autophagy markers p62, LC3II/LC3I, and Beclin-1, as well as the mitophagy kinases PINK1 and Parkin, in D-galactose-induced mouse heart. As depicted in Figure 5, Western blotting results showed that in the D-galactose group, the p-ULK1/ULK1 ratio was decreased, the expression of the key autophagy protein p62 was increased, and the expression levels of LC3II/LC3I and Beclin-1 were both reduced. After separating the cytoplasm and mitochondria, the expression levels of PINK1 and Parkin in the cytoplasmic-enriched fraction of D-galactose group were significantly decreased, and thep-Parkin^S65^/Parkin ratio in the mitochondria-enriched fraction was also reduced. In contrast, after SGF treatment, p-ULK1/ULK1 ratio and the expression levels of the key autophagy proteins LC3II/LC3I and Beclin-1 were increased, while p62 protein expression was decreased; the expression levels of PINK1 and Parkin in the cytoplasm-enriched fraction, as well as p-Parkin^S65^/Parkin ratio in the mitochondria-enriched fraction, were all significantly increased. These results suggest that SGF exerts a protective effect against D-galactose-induced cardiac alterations in mice, and that its molecular mechanism may be mediated through the AMPK/ULK1-mediated PINK1/Parkin signaling pathway.

### 2.7. SGF Attenuated D-Galactose-Induced Senescence-like Changes in H9c2 Cells in an AMPK-Associated Manner

To more precisely elucidate the cardioprotective mechanism of SGF, we performed in vitro experimental validation. Figure 6 shows the effects of SGF on D-galactose-induced senescence-like changes in H9c2 cells, as detected by senescence-associated β-galactosidase (SA-β-gal) staining and Western blot analysis of the aging marker proteins p16 and p21. Compared with the control group, the percentage of SA-β-gal-positive (senescence) cells was increased by D-galactose in the D10 group, and the aging marker proteins p16 and p21 were also upregulated. The SGF treatment group could effectively mitigate these alterations, especially in the SGF-H group. Then, AMPK inhibitors-Compound C (CC) and AMPK agonist-AICAR were used to examine whether the effects of SGF are related to AMPK. The experimental results demonstrated that treatment with the AMPK-inhibitor CC inhibited AMPK phosphorylation, decreased the p-AMPKα/AMPK ratio, and significantly abolished the protective effect conferred by SGF-H. In contrast, AMPK agonist-AICAR produced a positive effect, and SGF-H and AICAR exhibited similar efficacy, both significantly blocking D-galactose-induced senescence-like changes in H9c2 cells. Collectively, these findings suggest that the positive effects produced by SGF in the D-galactose-induced H9c2 model is associated with AMPK activation.

### 2.8. SGF Inhibited ROS Overexpression in H9c2 Cells Induced by D-Galactose in an AMPK-Associated Manner

As shown in Figure 7, the D10 group exhibited a significant increase in ROS levels relative to the control group. In contrast, both the SGF-treated and AICAR-treated groups demonstrated marked suppression of ROS overexpression. Notably, co-administration of CC attenuated the ROS-suppressive effect of SGF.

### 2.9. SGF Upregulated the Expression of Key Factors in the PGC-1α/NRF1/TFAM Axis in D-Galactose-Induced H9c2 Cells in an AMPK-Associated Manner

We investigated the effect of SGF on the expression of key factors in the PGC-1α/NRF1/TFAM axis in a D-galactose-induced H9c2 cell senescence model in vitro, as shown in Figure 8. Compared with the control group, the mitochondria of cells stimulated by D-galactose exhibited similar structural damage as in the in vivo experiment, and the protein expression of key factors in the PGC-1α/NRF1/TFAM axis was significantly decreased. After pre-treatment with SGF, the expressions of NRF1, PGC1-α, and TFAM all significantly increased, which was similar to the effect of using AICAR. However, when Compound C, an AMPK inhibitor, was used, the recovery effects produced by SGF were all blocked, indicating that SGF upregulates the PGC-1α/NRF1/TFAM axis in an AMPK-associated manner.

### 2.10. SGF Upregulated the Expression of Key Factors in the PINK1/Parkin Signaling Pathway in D-Galactose-Induced H9c2 Cells in Association with AMPK/ULK1 Signaling

Finally, we examined whether SGF regulated PINK1/Parkin signaling pathway in association with AMPK/ULK1 signaling. Compared with the control group, JC-1 staining analysis demonstrated a significant reduction in mitochondrial membrane potential (ΔΨm) in the D10 group (Figure 9A,B), suggesting impaired mitochondrial function. Additionally, the expression of the autophagy protein p62 was increased, and the expression levels of LC3II/LC3I and Beclin-1 were decreased. However, after pre-treatment with SGF, especially the SGF-H group, an increase in mitochondrial membrane potential was observed, the expression of the autophagy protein p62 in cells was decreased, while the expression levels of LC3II/LC3I and Beclin-1 were increased (Figure 9E–H). In addition, we found that the fluorescence after MDC staining was enhanced, and the yellow fluorescence after Ad-mCherry-GFP-LC3B adenovirus transfection was also enhanced, indicating that SGF can affect D-galactose-induced autophagy in H9c2 cells. Then we detected that SGF increased p-ULK1/ULK1 ratio, the expressions of PINK1 and Parkin in the cytoplasm fraction significantly increased, the expressions of PINK1 and the p-Parkin^S65^/Parkin ratio in the mitochondria fraction also increased (Figure 9I–N). In addition, following CCCP treatment, the Mtphagy dye fluorescence signal was further enhanced during mitochondrial–lysosomal fusion in the SGF-treated H9c2 cells, the data results were similar to those using AICAR. In contrast, treatment with Compound C significantly reduced ΔΨm, the ratio of p-ULK1/ULK1, and the expression of key factors in the PINK1/Parkin signaling pathway. Therefore, the above data suggest that SGF may regulate the PINK1/Parkin signaling pathway via an AMPK/ULK1-associated mechanism, thereby potentially promoting mitophagy.

## 3. Discussion

Cardiovascular disease is the leading cause of death worldwide, and aging is a major risk factor for cardiovascular disease in the elderly population. There is a growing demand for anti-aging strategies and therapeutic approaches aimed at improving health and extending lifespan [40]. In this study, senescence-like models were established in mice and H9c2 cells using D-galactose. The cardioprotective effects of SGF were systematically evaluated in this model, revealing that SGF significantly attenuated D-galactose-induced cardiac alterations, inhibited myocardial hypertrophy, and preserved mitochondrial structural integrity. Mechanistic studies indicated that SGF activated AMPKα phosphorylation and, in an AMPK-associated manner, upregulated the expression of key proteins in the PGC-1α/NRF1/TFAM axis and the PINK1/Parkin signaling pathway, which may exert positive effects on mitochondrial biogenesis and mitophagy, thereby improving mitochondrial function and protecting the heart. These findings suggest that SGF may serve as a potential therapeutic candidate for cardiac aging, although further studies are warranted to validate its effects in other aging models and in humans.

D-galactose is an aldohexose and a commonly used inducer of accelerated aging models. Chronic high-dose administration of D-galactose produces oxidative stress and glycation damage in vivo, thereby inducing, within a relatively short period (weeks to months), myocardial structural and functional decline resembling natural aging [41]; it has therefore been widely used in cardiac aging research to recapitulate multiple core features of natural aging. In this study, we found that compared with the control group, D-galactose-treated mice showed pronounced left ventricular myocardial hypertrophy on echocardiographic parameters; significantly upregulated expression of hypertrophy-related genes (ANP, BNP, and β-MHC) and of the senescence markers p21 and p16 in myocardial tissue; and elevated levels of myocardial injury markers (NT-proBNP, cTnT, CK-MB, and CRP). Histopathological analysis revealed interstitial collagen deposition, enlarged cardiomyocyte cross-sectional area, and heightened ROS production in myocardial tissue. Collectively, these findings reflected a coordinated cascade of structural, biochemical, and molecular alterations characteristic of the heart in the D-galactose-induced mouse model, consistent with previous experimental studies [42]. Notably, treatment with SGF markedly attenuated all aforementioned pathological and molecular changes, suggesting that SGF exerts a protective effect against D-galactose-induced cardiac changes and may have potential interventional value for aging-related cardiovascular diseases. AMPK serves as a central metabolic regulator critical for maintaining cellular energy homeostasis under stress conditions. In cardiac tissue, AMPK activity exhibits a progressive decline with advancing age. However, the precise molecular mechanisms responsible for this age-associated reduction in AMPK activity remain incompletely characterized [30]. AMPK is a central regulatory node in mitochondrial quality control, coordinating mitochondrial health at multiple levels, from biogenesis and dynamics (fusion/fission) to autophagy/mitophagy [43]. The PGC-1α/NRF1/TFAM axis is a classic core pathway of mitochondrial biogenesis, with PGC-1α acting as the “master switch” of mitochondrial biogenesis, AMPK is the upstream regulatory target acting on PGC-1α. Under normal circumstances, AMPK phosphorylation directly upregulates PGC-1α expression, initiating a downstream cascade that promotes the expression of NRF1/2, induces the expression of TFAM and transfers it to the mitochondria, where it binds to mitochondrial DNA, activating transcription and replication, ultimately enhancing mitochondrial biogenesis and functional capacity [44]. Notably, PGC-1α—beyond its well-established role in mitochondrial biogenesis—is critically involved in the regulation of oxidative stress and serves as a central modulator of mitochondrial redox biology and ROS homeostasis [44,45]. In the D-galactose-induced mouse model, we found that SGF ameliorated mitochondrial structural abnormalities, upregulated AMPKα phosphorylation and the protein expression of key factors in the PGC-1α/NRF1/TFAM axis in mouse heart, and additionally suppressed excessive ROS production in myocardial tissue. In the in vitro experiments, we found that AMPK inhibitor compound C abolished the effects of SGF and suppressed the PGC-1α/NRF1/TFAM axis, whereas activation of AMPKα phosphorylation with AICAR upregulated the expression of key factors in the PGC-1α/NRF1/TFAM axis. As a well-characterized AMPK activator, AICAR stimulation increases AMPKα phosphorylation and upregulates PGC-1α and its downstream targets NRF1 and TFAM, thereby further promoting mitochondrial biogenesis [46]; it can also regulate the PINK1/Parkin pathway through AMPK and influence mitophagy [47]. The effects of SGF closely resemble those of AIACR. We therefore speculated that AMPKα may be the primary molecular target through which SGF regulated the PGC-1α/NRF1/TFAM axis, and that SGF may suppress excessive ROS production via AMPK/PGC-1α.

Autophagy is a crucial mechanism for maintaining cellular homeostasis and function. It selectively targets damaged or superfluous proteins, nucleic acids, and organelles for lysosomal degradation, thereby facilitating the recycling of amino acids to support energy metabolism and new enzyme synthesis. Mitophagy, a selective form of autophagy, specifically recognizes and eliminates dysfunctional mitochondria to preserve mitochondrial quality and cellular bioenergetic integrity. The PINK1/Parkin signaling pathway is the most representative among mitophagy pathways, including Parkin, PINK1, Beclin-1, LC3, and p62, etc. In summary, mitochondrial damage is detected through a decline in ΔΨm, resulting in mitochondrial depolarization. In the cytoplasm, PINK1 accumulates on the outer membrane of the damaged mitochondria and is activated. Subsequently, Parkin is recruited from the cytoplasm to the mitochondrial surface and its E3 enzyme activity is activated, causing the outer mitochondrial membrane proteins to be ubiquitinated, and recruiting autophagy-related proteins (such as p62 and LC3), initiating the formation of autophagosomes that engulf the damaged mitochondria and transfer them to lysosomes for degradation [48,49]. Beclin-1 is a key regulatory factor that recruits autophagy-related proteins to the autophagy precursor, and it can regulate autophagy activity [50]. During autophagy, the cytoplasmic LC3 (LC3I) peptide is partially hydrolyzed to form the membrane-bound LC3 (LC3II); the LC3II/LC3I ratio or absolute LC3II levels are widely used as quantitative indicators of autophagy. The p62 protein serves as a bridge between LC3 and ubiquitinated proteins, and is selectively encapsulated into autophagosomes for degradation. Consequently, p62 levels inversely correlate with autophagic activity: they decrease during active autophagy and accumulate upon autophagic inhibition; p62 is also regarded as a biomarker of autophagy [51].

ULK1 is one of the core genes related to autophagy and serves as a direct phosphorylation substrate of AMPK. It has been reported that AMPK/ULK1 interacts with the PINK1/Parkin signaling pathway and plays a crucial role in mitochondrial autophagy. Indeed, cell lines deficient in ULK1 or AMPK displayed a significant accumulation of morphologically aberrant mitochondria, underscoring the essential role of ULK1 and AMPK phosphorylation in mediating mitophagy [52,53]. In this study, we found that, in cardiac tissue, D-galactose not only inhibited AMPK phosphorylation but also suppressed ULK1 phosphorylation accompanied by increased p62 expression, a decreased LC3II/LC3I ratio, and reduced Beclin-1 expression. More importantly, in the mitochondrial-enriched fraction, the ratio of p-Parkin^S65^/Parkin was also significantly decreased. Notably, treatment with SGF effectively reversed these alterations. Subsequently, in in vitro experiments, it was further discovered that SGF treatment effectively reversed the D-galactose-induced decline in mitochondrial membrane potential (ΔΨm), indicating an improvement in the mitochondrial energized state. It also activated AMPK and ULK1 phosphorylation, upregulated LC3II/LC3I and Beclin-1 expression, and altered p62 abundance. Using MDC staining and Ad-mCherry-GFP-LC3B transfection, we further found that the fluorescence images obtained were consistent with the changes in autophagy-associated markers. Moreover, in the mitochondrial-enriched fraction of H9c2 cells, the ratio of p-Parkin^S65^/Parkin was significantly increased. Relevant studies have pointed out that the phosphorylation of Parkin at Ser65 is crucial for the activation of Parkin within the cell and its participation in mitophagy [54]; this evidence was consistent with the increase in mitophagy fluorescence expression observed using the Mtphagy dye. However, treatment with the AMPK inhibitor compound C reversed the observed effect, whereas administration of AICAR enhanced the fluorescence signal. These changes suggested that SGF could modulate the expression of key factors in the PINK1/Parkin signaling pathway in an AMPK/ULK1-associated manner, potentially exerting a positive effect on mitophagy. Most studies indicated that, during PINK1/Parkin-dependent mitophagy, PINK1 phosphorylate Parkin Ser65 and ubiquitin Ser65 were key to the activation of parkin, which ubiquitinates mitochondrial proteins and thereby initiates the process of mitophagy [39,55]; this was also confirmed by our research results. But the relationship between AMPK and mitochondrial function remains intricate. Studies have shown that PINK1 phosphorylation of Parkin at Ser65 is contingent upon prior ULK1 phosphorylation of Parkin at Ser108, which plays an important role in the early stage of mitophagy [56]. Moreover, there may be other regulatory factors between ULK1 and PINK1/Parkin pathway, which have not been clarified in this study, and these will also be the focus of our future research.

There are some limitations in our study. Although our findings established the overall protective efficacy of the SGF extract and confirmed its modulatory effects on two key pathways of mitochondrial biogenesis and mitophagy (the PGC-1α/NRF1/TFAM axis and the PINK1/Parkin signaling pathway), this study focused only on the role of AMPK and provides only preliminary insight into its potential mechanisms of action. In addition, without an osmotic control, the acute stress component cannot be fully distinguished from the senescence-associated changes; the observed cellular phenotype should therefore be interpreted as senescence-like rather than as established senescence. Specifically, SGF increased the expression of key proteins related to the PGC-1α/NRF1/TFAM axis and the PINK1/Parkin signaling pathway. However, the current data only suggest that AMPK may be involved. It is well known that the α subunit is the catalytic core of AMPK, but the α subunit is further divided into α1 and α2, in different species and pathological states; their expression ratios and functional weights in the heart also differ [57,58]. We have not yet determined which isoform (AMPKα1 or α2) is responsible for the AMPK activity. Moreover, the pharmacological tools used (Compound C and AICAR) have inherent limitations, including AMPK-independent effects, and it has not yet been demonstrated that AMPK is required for the effects of SGF. Other potential molecular targets or complementary signaling pathways may be involved, and future experiments using gene knockout may be needed to precisely elucidate the regulatory mechanisms of SGF. Furthermore, SGF constitutes a mixture of multiple flavonoids; thus, it is currently unclear whether its cardioprotective effects arise from synergistic multi-target interactions among these constituents or are attributable to a single predominant flavonoid. We will conduct further research and verification in the future to determine this.

## 4. Materials and Methods

### 4.1. Smilax glabra Roxb. Flavonoids (SGF) Extraction

The dried rhizome of *Smilax glabra* Roxb. (lot number: 20220901) was purchased from Zhejiang Zoli Baicao Traditional Chinese Medicine Ltd. (Huzhou, Zhejiang, China). Prior to extraction, the rhizomes were comminuted into uniform particles (20–60 µm), and then the material was extracted with 80% ethanol (material-to-liquid ratio 1:15), with flash extraction (100 V for 90 s); this process was repeated three times. After filtration, the extract solution was collected and ethanol was recovered to obtain the crude extract of SGF. An appropriate amount of water was added to disperse the crude extract of SGF, and then it was purified using HP-100 macroporous resin. The purified SGF was concentrated and evaporated again, then freeze-dried to form powder which was stored in a desiccator. Using rutin as the standard, the total flavonoid content of SGF was determined to be 71.05% (expressed as rutin equivalents) by the colorimetric method. The high-performance liquid chromatography (HPLC) (Agilent 1260 Infinity III, Agilent, San Jose, CA, USA) was used to detect the content of astilbin in SGF for quality control. The HPLC conditions were as follows: Column: YMC-pack ODS-A (250 × 4.6 mm, 5 μm); Mobile phase: Methanol–0.1% glacial acetic acid (40:60); Wavelength: 290 nm; Flow rate: 1.0 mL/min; Column temperature: 25 °C. The percentage content of astilbin in SGF was 18.92%, as shown in Figures S1 and S2.

### 4.2. Animals Treatment

Thirty-two male C57BL/6J mice (age, 6–8 weeks; weight, 18–20 g) were purchased from Zhejiang Vital River Laboratory Animal Technology Co., Ltd. (Jiaxing City, Zhejiang Province, China) (SCXK 2024-0001), and were maintained in the Department of Experimental Animals, Zhejiang Academy of Traditional Chinses Medicine (SYXK 2024-0010). The animals were maintained under a 12 h light/dark cycle at 22 ± 2 °C and 50 ± 10% relative humidity. Standard rodent chow and sterilized water were available ad libitum. All experimental procedures were carried out in strict accordance with China’s legislation on the use and care of experimental animals, and the study protocol received ethical approval from the Institutional Animal Care and Use Committee (IACUC Approval No. PRO 2023-016).

Thirty-two male C57BL/6J mice were randomly divided into 4 groups (*n* = 8): control group (sterile water + normal saline), D-galactose (G0750, Sigma-Aldrich, St. Louis, MO, USA) group (sterile water +150 mg/kg/day D-galactose), SGF low-dose group (78 mg/kg/day SGF +150 mg/kg/day D-galactose), and SGF high-dose group (156 mg/kg/day SGF +150 mg/kg/day D-galactose); SGF was dissolved in sterile water, and D-galactose was prepared in normal saline. Subcutaneous injection of D-galactose/normal saline (0.1 mL/10 g) was performed every day between 8 and 9 a.m., and oral gavage of SGF/sterile water (0.1 mL/10 g) was performed between 5 and 6 p.m. Mice in the D-galactose group received subcutaneous injections of D-galactose solution for 12 consecutive weeks. In the SGF treatment group, oral SGF administration was started after 4 weeks of subcutaneous D-galactose injection, during which subcutaneous D-galactose injections continued; SGF treatment lasted 8 weeks, as shown in Figure 10.

### 4.3. Echocardiography Examination

Cardiac echocardiography was performed every 4 weeks using a high-resolution small animal ultrasound imaging system (ZS3 SCI, Mindray, Shenzhen, Guangdong, China). Mice were anesthetized with isoflurane gas (induction at 3–4% and maintenance at 1.5–2% in oxygen) and placed in a supine position on a heating pad. M-mode echocardiograms were recorded at the level of the mitral valve tendons. The left ventricular end-diastolic interventricular septal thickness (IVSd), left ventricular end diastolic diameter (LVIDd), left ventricular end-diastolic posterior wall thickness (LVPWd), left ventricular end-systolic interventricular septal thickness (IVSs), left ventricular end-systolic diameter (LVIDs), and left ventricular end-systolic posterior wall thickness (LVPWs) were measured. The ejection fraction (EF), fraction shortening (FS), left ventricular end-diastolic volume (LVEDV), and left ventricular end-systolic volume (LVESV) were calculated. Each index was measured on 3 consecutive cardiac cycles and the average value was taken.

### 4.4. Heart Weight Assessment and Histological Examination

After the experiment, weigh the mice, obtain their hearts after anesthesia, weigh the hearts, and calculate the heart weight-to-body weight ratio. The non-apical part of the mouse hearts was fixed in 10% neutral formaldehyde for 24 h, then dehydrated with alcohol and embedded in paraffin. The samples were cut into 4 μm thin slices using a slicer, and then stained with Hematoxylin–Eosin (H&E) for histopathological examination; Masson’s trichrome staining was used to assess collagen deposition, and wheat germ agglutinin (WGA) staining was used to evaluate the size of myocardial cells. The Image-Pro Plus 6.0 software (Image Pro Plus Software, Rockville, MD, USA) was used to assess the degree of myocardial tissue fibrosis (the blue positive area of collagen fibers/total myocardial tissue area × 100%) and the area of myocardial cells for each sample.

### 4.5. Enzyme-Linked Immunosorbent Assay (ELISA)

The mice were fasted for 12 h and allowed to drink freely. After anesthesia, 0.5 mL of blood samples were collected and centrifuged at 3000× *g* for 15 min at 4 °C. The concentrations of NT-proBNP (MB-3294B, Jiangsu Meibiao Biotechnology, Nanjing, Jiangsu, China), cTnT (MB-7278B, Jiangsu Meibiao Biotechnology, Nanjing, Jiangsu, China), CK-MB (MB-5947B, Jiangsu Meibiao Biotechnology, Nanjing, Jiangsu, China), and CRP (MB-2810B, Jiangsu Meibiao Biotechnology, Nanjing, Jiangsu, China) were determined using an ELISA kit according to the manufacturer’s instructions. All serum samples were diluted 5-fold with the provided sample diluent before measurement of CRP and cTnT. Concentrations were calculated by multiplying the assay reading by the dilution factor (×5). After dilution, all assay readings fell within the standard curve range of the respective kits. The absorbance at 450 nm was measured using a microplate reader (Varioskan Flash, ThermoFisher, Waltham, MA, USA).

### 4.6. Quantitative Real-Time Polymerase Chain Reaction (qRT-PCR)

The total RNA of mouse myocardial tissue was extracted using the Trizol method. After determining the concentration and purity of the total RNA, it was reverse-transcribed into cDNA. The cDNA was then subjected to real-time PCR amplification using a real-time fluorescence quantitative PCR instrument (Applied Biosystems, ThermoFisher, Waltham, MA, USA), with the reference gene used as a standard for relative quantification. The relative expression levels of ANP, BNP, β-MHC and the housekeeping gene GAPDH were calculated using the 2^−ΔΔCt^ method.

The primer sequences used in this study are listed as follows: forward ANP, (5′–3′) GTGCGGTGTCCAACACAGAT and reverse primer, (5′–3′) TCCAATCCTGTCAATCCTACCC; forward BNP, (5′–3′) GAGGCGAGACAAGGGAGAAC and reverse primer, (5′–3′) CCTTCAGGAGATCCATGCCG; forward β-MHC, (5′-3′) ACTGTCAACACTAAGAGGGTCA and reverse primer, (5′–3′) TTGGATGATTTGATCTTCCAGGG; forward GAPDH, (5′–3′) CCTCGTCCCGTAGACAAAATG and reverse primer, (5′–3′) TGAGGTCAATGAAGGGGTCGT.

### 4.7. Cell Group and Treatment

H9c2 embryonic rat-heart-derived myoblasts were obtained from Stem Cell Bank, Chinese Academy of Sciences (Shanghai, China). Cells were cultured in DMEM containing 10% fetal bovine serum, penicillin (100 U/mL), and streptomycin (100 µg/mL) at 37 °C under 5% CO_2_.

H9c2 cells were seeded into 6-well plates and randomly divided into the following groups: control group, D10 group (10 g/L D-galactose), D10+SGF-Lgroup (10 g/L D-galactose + 62.5 μg/mL SGF), and D10+SGF-H group (10 g/L D-galactose + 125 μg/mL SGF). In the SGF treatment group, SGF was added 30 min before D-galactose stimulation and co-cultured for 48 h.

To further clarify whether SGF exerts a cardiac protective effect by activating AMPK, AMPK agonist-AICAR (S1802, Selleck, Houston, TX, USA) and AMPK inhibitor-Compound C (HY-13418A, MedChem Express, Monmouth Junction, NJ, USA) were added to the cells, which were divided into the following groups: D10+SGF-H+CC group (10 g/L D-galactose + 125 μg/mL SGF+ 15 µM AMPK inhibitor-Compound C), D10+ AICAR group (10 g/L D-galactose + 1mM AMPK agonist-AICAR). Each group was incubated for 48 h in three independent biological replicates (*n* = 3), and each was seeded from a separately prepared culture.

### 4.8. Cellular Senescence-Associated β-Galactosidase (SA-β-Gal) Staining

According to the instructions of the kit (C0602, Beyotime, Shanghai, China), the culture medium in the wells was removed, and then washed once with PBS or HBSS. After that, 1 mL of β-galactosidase staining fixation solution was added and the cells were fixed at room temperature for 15 min. Then, the fixation solution was removed, and the cells were washed three times with PBS or HBSS. Subsequently, 1 mL of the prepared staining working solution was added to each well, and the cells were incubated at 37 °C overnight. The 6-well plate was sealed with parafilm or plastic wrap to prevent evaporation, and then observed under a common optical microscope for the staining results.

### 4.9. Monodansylcadaverine (MDC) Staining

MDC staining was used as an autophagosome tracer to detect autophagy. After grouping the cells, each group was added with MDC staining solution (C3018S, Beyotime, Shanghai, China), which was incubated in a cell culture box at 37 °C in the dark for 30 min. The cells were then washed three times with detection buffer and observed under a fluorescence microscope.

### 4.10. Mitochondrial Membrane Potential Measurement

H9c2 cells were seeded in six-well plates, subjected to grouped treatments, after which the culture medium was aspirated. The cells were then washed with PBS, according to the instructions of the kit (C2003S, Beyotime, Shanghai, China); 1 mL of cell culture medium and 1 mL of JC-1 staining working solution were added. The cells were incubated in a cell culture incubator at 37 °C for 20 min. Then, the supernatant was aspirated, washed with JC-1 staining buffer, and then 2 mL of cell culture medium was added before observation under a fluorescence microscope.

### 4.11. m-Cherry GFP LC3B Transfection

According to the manufacturer’s instructions, the autophagy process of infected cells was detected using Ad-mCherry-GFP-LC3B (C3011, Beyotime, Shanghai, China). H9C2 cells were seeded and infected with the Ad-mCherry-GFP-LC3B adenovirus vector (MOI = 40). Twenty-four hours after viral infection, the virus-containing culture medium was removed and fresh culture medium was added for further cultivation for 24 h. Then, the cells were grouped and the fluorescence expression levels of each group were observed under a confocal microscope.

### 4.12. Mitophagy Assay

H9c2 cells were seeded into confocal dishes. After treatment with D-galactose and SGF according to the grouping, the medium was discarded, and the cells were washed twice with serum-free medium according to the manufacturer’s instructions. Then, the prepared Mtphagy Dye (MD01, Dojindo, Kumamoto, Japan) working solution was added, and the cells were cultured at 37 °C for 30 min. The cells were washed twice again with serum-free medium, followed by the addition of a mitophagy inducer (10 µmol/L CCCP for 24 h of incubation). Subsequently, the prepared Lyso Dye working solution was added, and the cells were cultured at 37 °C for another 30 min. After washing the cells twice with serum-free medium, fluorescence expression was observed using a confocal microscope.

### 4.13. Electron Microscopy

Fresh myocardial tissue was cut into 1 mm × 1 mm × 1 mm tissue blocks and fixed in 2.5% glutaraldehyde at 4 °C for more than 6 h. For the H9C2 cells, after aspirating the culture medium, 4 °C 2.5% glutaraldehyde was added, followed by rapid scraping of the cells with a cell scraper. After centrifugation to remove the supernatant, 4 °C 2.5% glutaraldehyde was added again for fixation. These samples were fixed with 1% osmic acid at room temperature for 2–3 h, and then underwent dehydration, infiltration, embedding, sectioning, staining, etc., and then the mitochondrial morphology was observed with a transmission electron microscope.

### 4.14. Dihydroethidium (DHE) Staining

Using DHE staining to evaluate the reactive oxygen species (ROS) expression levels in myocardial tissue or cells, fresh heart tissue from mice was obtained, and the frozen myocardial tissue was sliced into 10 μm thick frozen sections, and H9C2 cells were seeded onto cell climbing slides. The DHE probe (S0063, Beyotime, Shanghai, China) was mixed with PBS buffer solution and then dropped onto the tissue sections or cell climbing slides, followed by incubation in the dark for 30 min to 1 h to ensure sufficient entry of DHE into the cells. After removing the DHE staining solution and thoroughly rinsing with PBS, the red fluorescence intensity was observed under a fluorescence microscope.

### 4.15. Western Blot Analysis

According to the instructions, use the mitochondrial isolation kit to obtain the cytoplasmic proteins and mitochondrial proteins from the tissues (C3606, Beyotime, Shanghai, China) and cells samples (C3601, Beyotime, Shanghai, China), then perform protein quantification using the BCA method. The total cytoplasmic protein was separated by 10% sodium dodecyl sulfate (SDS)-polyacrylamide gel electrophoresis and then transferred onto a polyvinylidene fluoride (PVDF) membrane. The membrane is incubated at room temperature in a blocking medium containing 5% BSA for 1 h, and then incubated overnight at 4 °C with one of the following primary antibodies targeting specific proteins. p21 (ab109199, Abcam, 1:1000), p16 (ab51243, Abcam, 1:5000), LC3 II/LC3 I(12741S, CST, 1:1000), p62 (ab109012, Abcam, 1:2000), Beclin-1(3738, CST, 1:1000), AMPKα (5831S, CST, 1:1000), p-AMPKα(Thr 172)(50081S, CST, 1:1000), ULK1(8054, CST, 1:1000), p-ULK1 (Ser555) (5869S, CST, 1:500), PGC-1α(ab191838, Abcam, 1:1000), NRF1(12482-1-AP, Proteintech, 1:1500), TFAM(MA516148, ThermoFisher, 1:1000), PINK1(sc-517353, Santa Cruz Biotechnology (Dallas, TX, USA), 1:1000), Parkin(sc-32282, Santa Cruz Biotechnology, 1:1000), β-actin(66009-1-Ig, Proteintech, 1:10000). After recovering the primary antibody, a secondary antibody was added for incubation for 0.5 h, and then detected using a chemiluminescence instrument.

Mitochondrial proteins were detected by Automatic Western blot quantitative analysis system (Jess, ProteinSimple, Minneapolis, MN, USA). According to the instructions of the reagent kit related to the instrument, the protein standard sample and mitochondrial protein sample were prepared. They were heated at 95 °C for 5 min and then cooled on ice for 5 min. Using the reverse-phase liquid absorption method, the mitochondrial protein sample, protein standard, secondary antibody, and primary antibody were added into the microplate. The required primary antibodies were as follows: PINK1(sc-517353, Santa Cruz Biotechnology, 1:500), Parkin (sc-32282, Santa Cruz Biotechnology, 1:500), p-Parkin (Ser65) (PA5114616, ThermoFisher, 1:1000), VDAC1(ab15895, Abcam, 1:1000). The microplate was centrifuged at 2500 r·min^−1^for 5 min at room temperature. After confirming that there were no bubbles at the bottom of the wells, the prepared microplate was placed in the instrument for the detection and analysis of the expression levels of each protein.

### 4.16. Statistical Analysis

All experimental data were processed using the GraphPad Prism 10.6 software (GraphPad Software, San Diego, CA, USA). The data were expressed as mean ± standard deviation. Normality and homogeneity of variance were checked using the Shapiro–Wilk test and Levene’s test, respectively. When these assumptions were met, comparisons among groups were made using one-way ANOVA followed by Tukey’s post hoc test for all pairwise comparisons, or by Dunnett’s post hoc test when each treatment group was compared only with the control group. When the assumptions were not met, the Kruskal–Wallis test followed by Dunn’s post hoc test was used. Multiplicity was controlled by the respective post hoc procedure. In cell experiments, “*n*” refers to the number of independent biological replicates. *p*-values of less than 0.05 were considered statistically significant.

## 5. Conclusions

In summary, our study shows that SGF exerts a protective effect against D-galactose-induced cardiac alterations, activates AMPK, and modulates the expression of key factors in the PGC-1α/NRF1/TFAM axis and the PINK1/Parkin signaling pathway, possibly thereby exerting positive effects on mitochondria to protect the heart. These findings suggest that SGF may have considerable potential in the treatment of aging and related cardiovascular diseases.

## Figures and Tables

**Figure 1:**
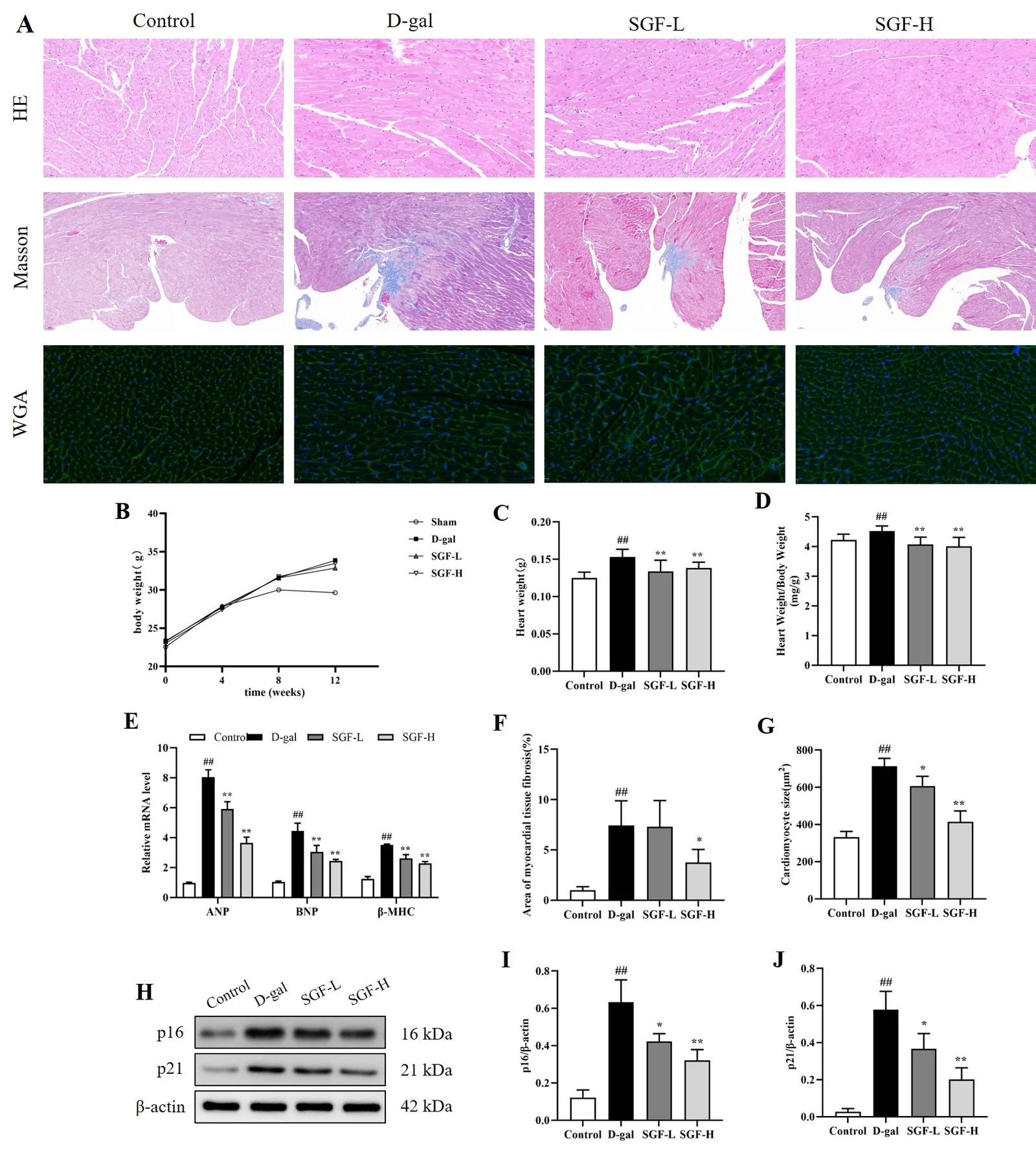
Effects of SGF on myocardial senescence biomarkers and histopathological alterations in D-galactose mice. (**A**) Representative views of hematoxylin and eosin (HE) and Masson’s trichromestaining (total magnification 200×), WGA (wheat germ agglutinin) staining of cross sections (total magnification 400×). (**B**–**D**) The body-weight curves of the mice over the experimental period, together with the heart weight(g) and the ratio of heart weight-to-body weight (mg/g) in each group after 12 weeks of D-galactose treatment, *n* = 8 for each group. (**E**) Relative expression levels of hypertrophic genes ANP, BNP and β-MHC, *n* = 4 for each group. (**F**,**G**) Quantitative analysis of myocardial fibrosis by Masson staining and the cross-sectional area of cardiomyocytes by WGA staining, *n* = 4 for each group. (**H**) Representative protein band images of p21 and p16. (**I**,**J**) Quantitative analysis of the aging markers p16 and p21, *n* = 4 for each group. ## *p* < 0.01 vs. control group, * *p* < 0.05, ** *p* < 0.01 vs. D-galactose group.

**Figure 2:**
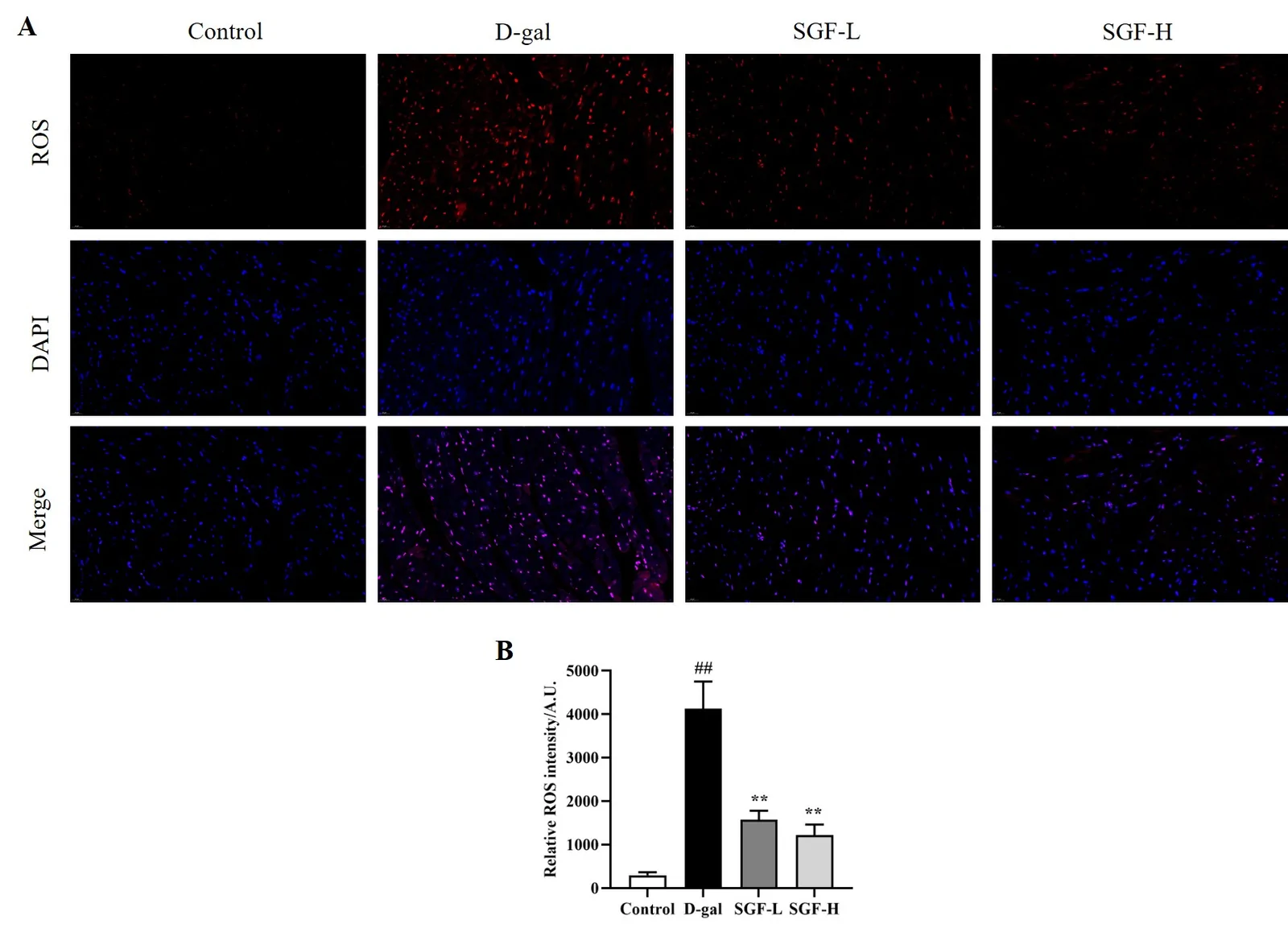
Effects of SGF on ROS expression in the myocardium of D-galactose mice. (**A**) Representative views of ROS fluorescence expression (total magnification 400×). (**B**) Quantitative analysis of ROS expression levels, *n* = 4 for each group. ## *p* < 0.01 vs. control group, ** *p* < 0.01 vs. D-galactose group.

**Figure 3:**
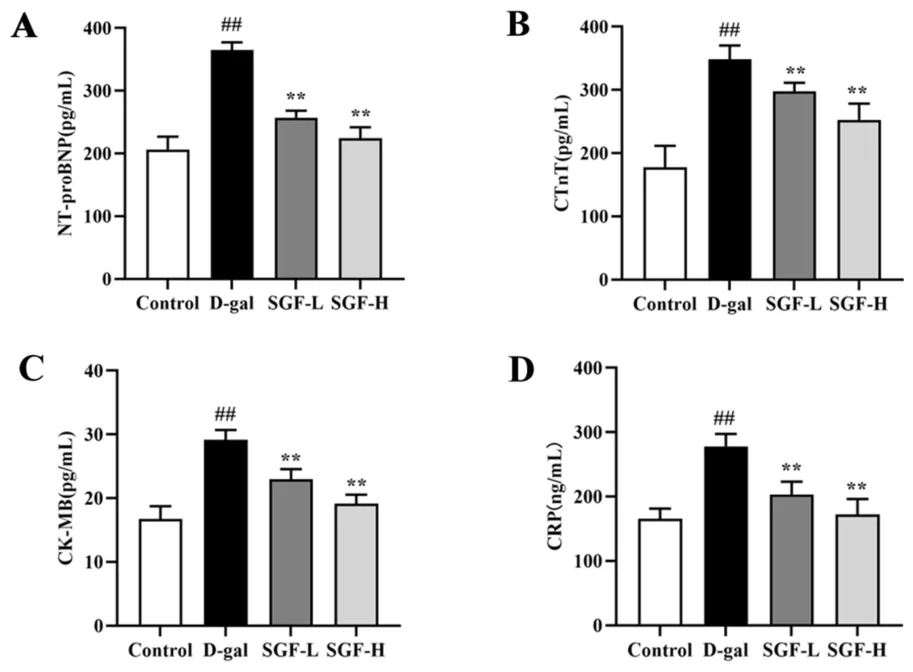
Effect of SGF on myocardial injury markers in serum of D-galactose mice. (**A**–**D**) Analysis of the contents of CTnT, CRP, NT-proBNP and CK-MB in mouse serum, *n* = 8 for each group. ## *p* < 0.01 vs. control group, ** *p* < 0.01 vs. D-galactose group.

**Figure 4:**
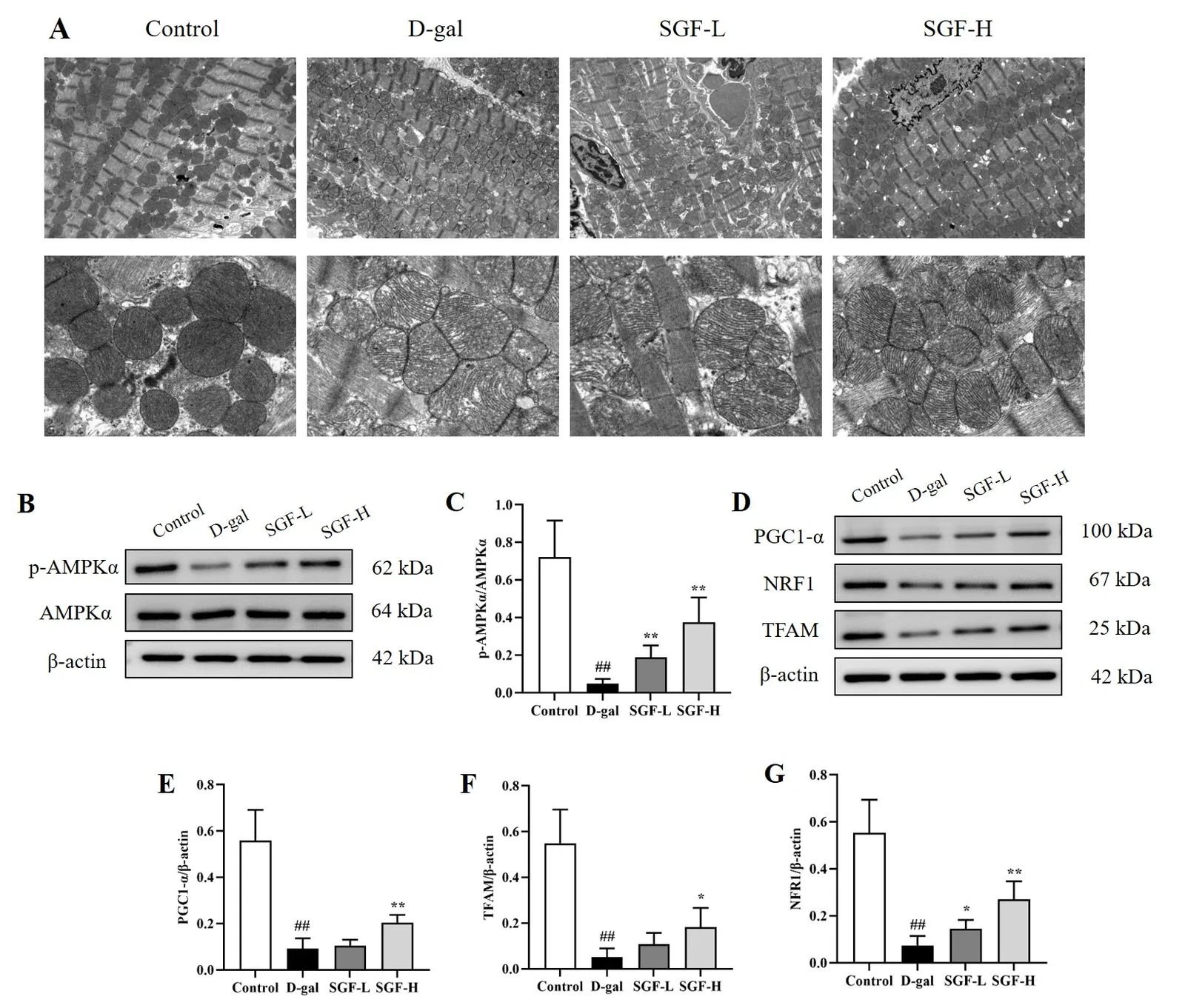
Effects of SGF on the expression of key factors in the AMPK and PGC-1α/NRF1/TFAM axis in D-galactose mice. (**A**) Transmission electron microscopy images of mitochondria in mouse cardiomyocytes (total magnification 2500×/10,000×), *n* = 4 for each group. (**B**,**C**) Quantitative analysis and representative bands of p-AMPKα and AMPKα. (**D**–**G**) Quantitative analysis and representative bands of PGC-1α, NRF1, and TFAM, *n* = 4 for each group. ## *p* < 0.01 vs. control group, * *p* < 0.05, ** *p* < 0.01 vs. D-galactose group.

**Figure 5:**
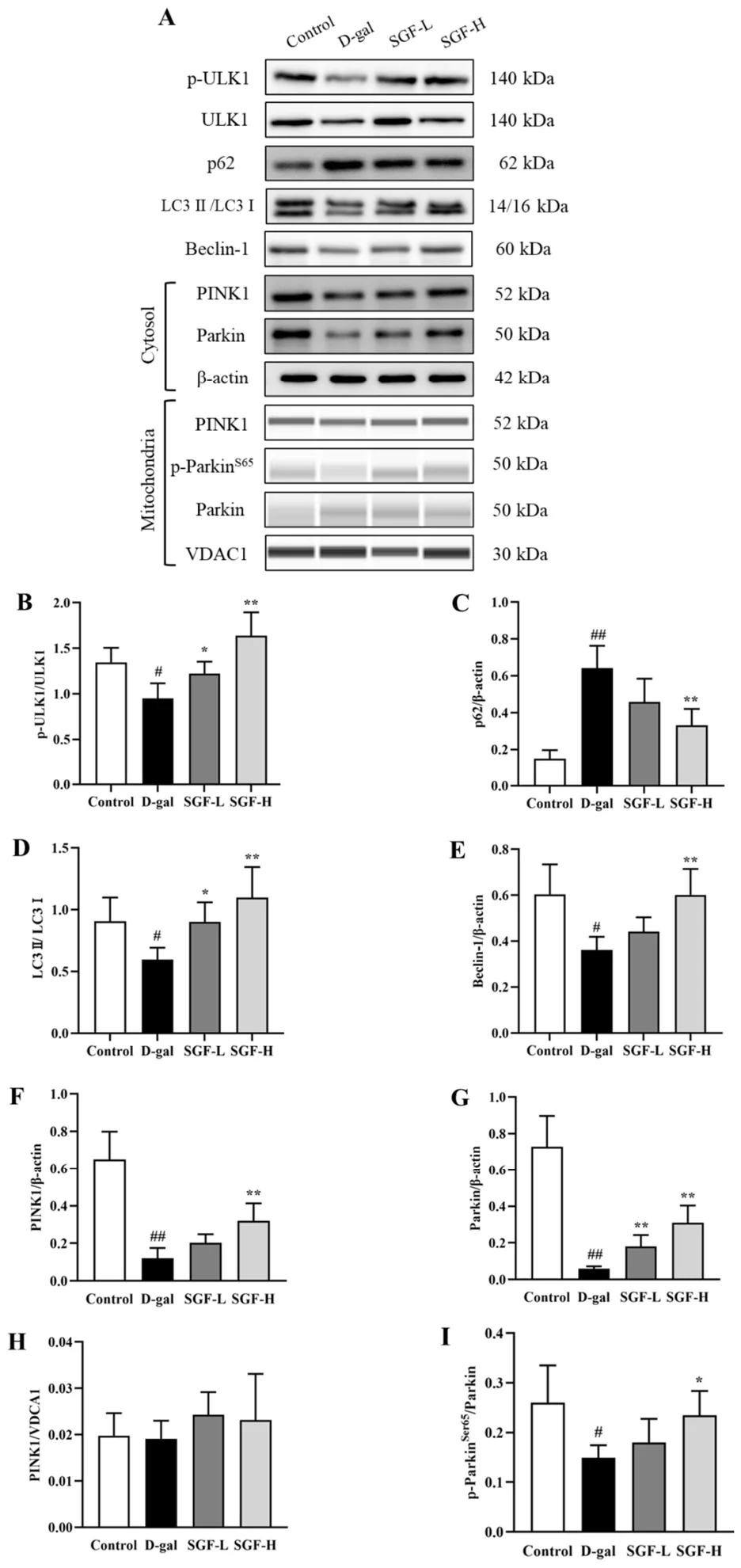
Effect of SGF on key factors of AMPK/ULK1-mediated PINK1/Parkin signaling pathway in D-galactose mice. (**A**) Representative protein band images of Western blots. (**B**–**I**) Quantitative analysis of p-ULK1, ULK1, autophagy-related proteins (p62, LC3II/LC3I, and Beclin-1) and proteins related to the PINK1/Parkin signaling pathway, *n* = 4 for each group. # *p* < 0.05, ## *p* < 0.01 vs. control group, * *p* < 0.05, ** *p* < 0.01 vs. D-galactose group.

**Figure 6:**
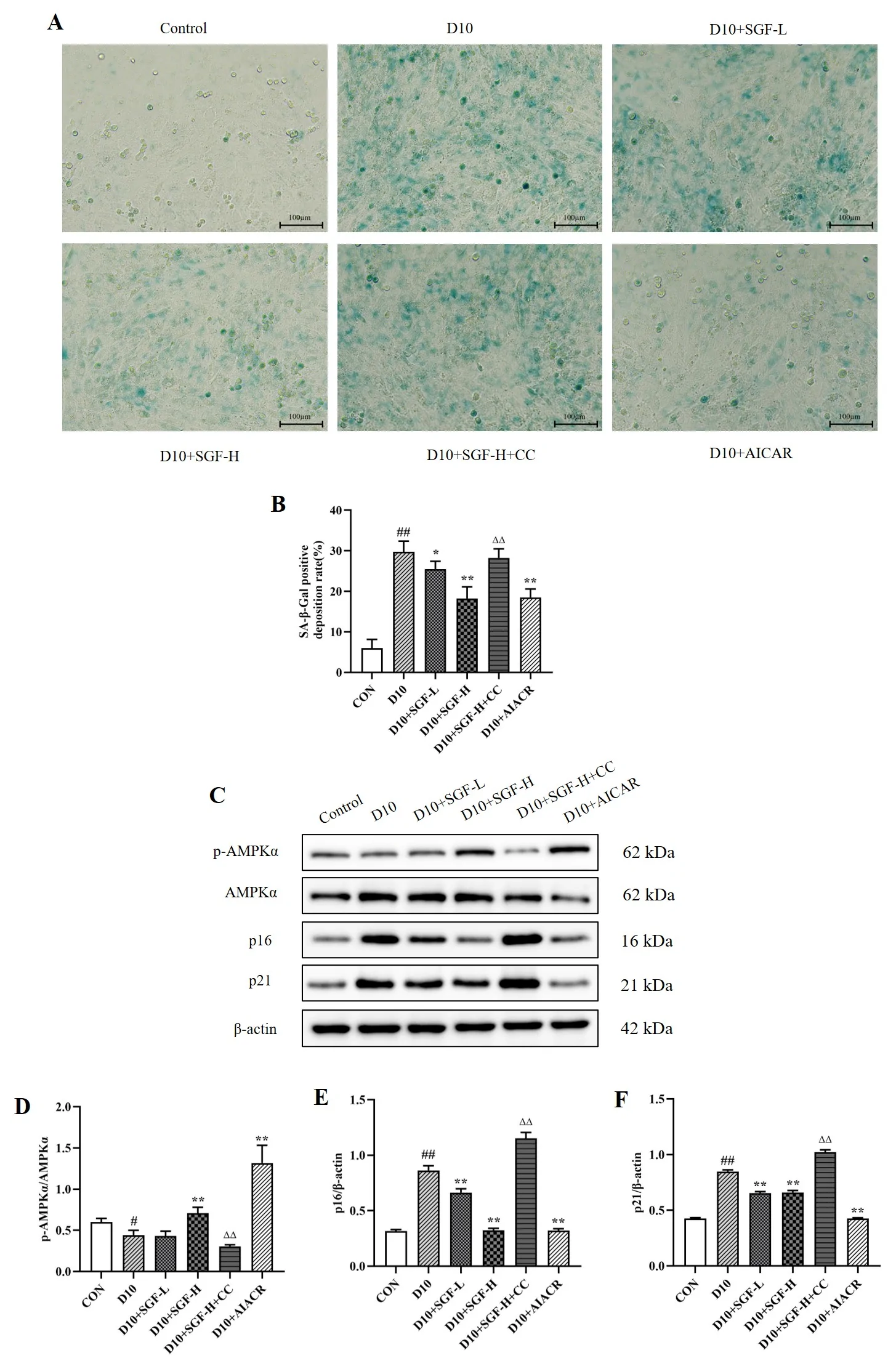
SGF attenuated D-galactose-induced senescence-like changes in H9c2 cells in an AMPK-associated manner. (**A**,**B**) Representative images and quantitative analysis of SA-β-gal aging staining, senescent cells are shown in blue (scale bars = 100 μm), *n* = 3 for each group. (**C**) Representative protein band images of p21, p16 and p-AMPKα/AMPKα. (**D**–**F**) Quantitative analysis of p21, p16 and p-AMPKα/AMPKα. *n* = 3 for each group. # *p* < 0.05, ## *p* < 0.01 vs. control group, * *p* < 0.05, ** *p* < 0.01 vs. D10 group, ^∆∆^ *p* < 0.01 vs. D10+SGF-H group.

**Figure 7:**
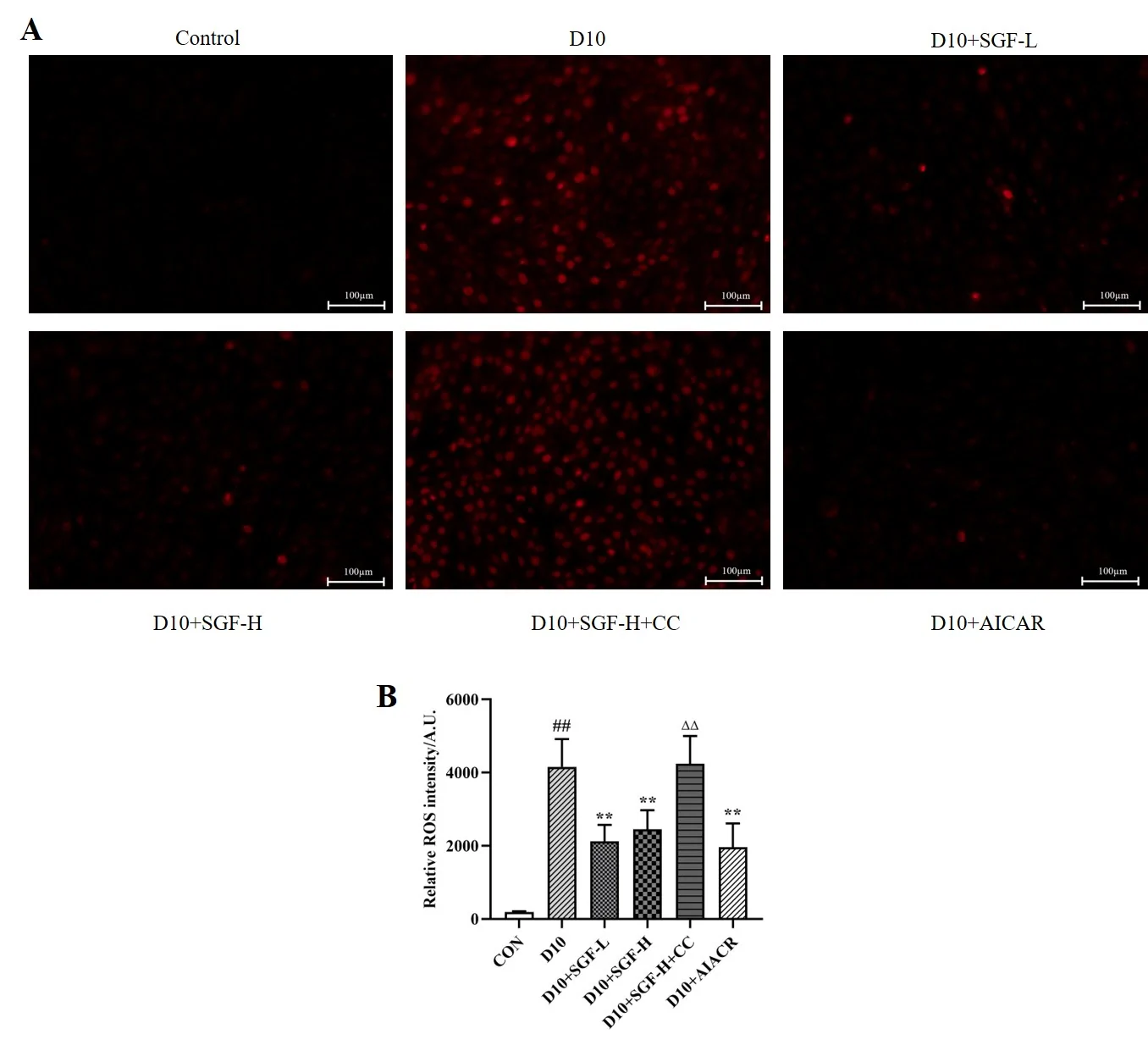
SGF inhibited ROS overexpression in H9c2 cells induced by D-galactose in an AMPK-associated manner. (**A**) Representative images of DHE staining (scale bars = 100 μm). (**B**) Quantitative analysis of DHE staining. *n* = 3 for each group. ## *p* < 0.01 vs. control group, ** *p* < 0.01 vs. D10 group, ^∆∆^ *p* < 0.01 vs. D10+SGF-H group.

**Figure 8:**
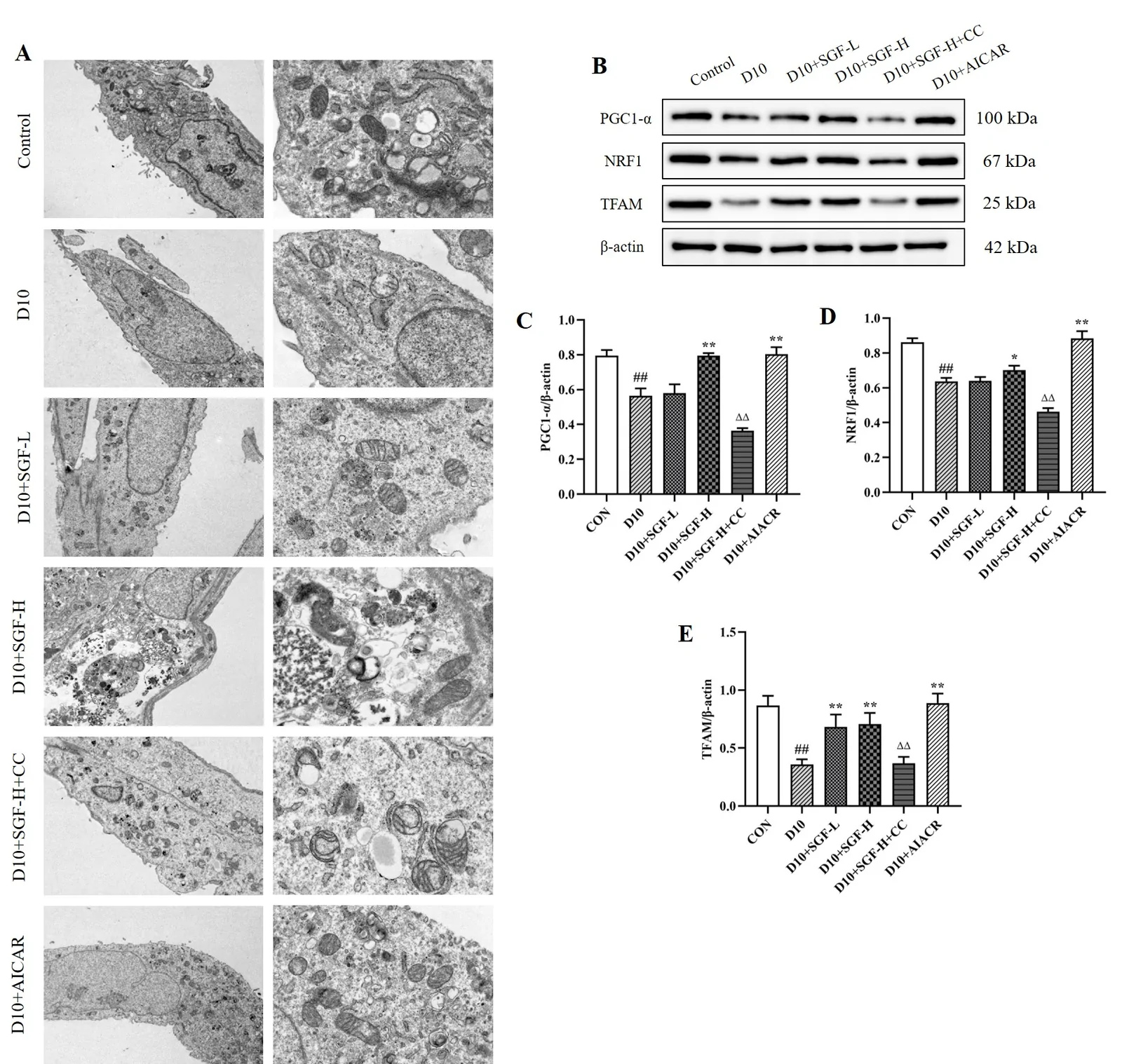
SGF upregulated the expression of key factors in the PGC-1α/NRF1/TFAM axis in H9c2 cells induced by D-galactose in an AMPK-associated manner. (**A**) Transmission electron microscopy images of mitochondria in H9c2 cells (total magnification 2500×/10,000×), *n* = 3 for each group. (**B**) Representative protein band images of Western blots. (**C**–**E**) Quantitative analysis of PGC1-α, NRF1, and TFAM, *n* = 3 for each group. ## *p* < 0.01 vs. control group, * *p* < 0.05, ** *p* < 0.01 vs. D10 group, ^∆∆^ *p* < 0.01 vs. D10+SGF-H group.

**Figure 9:**
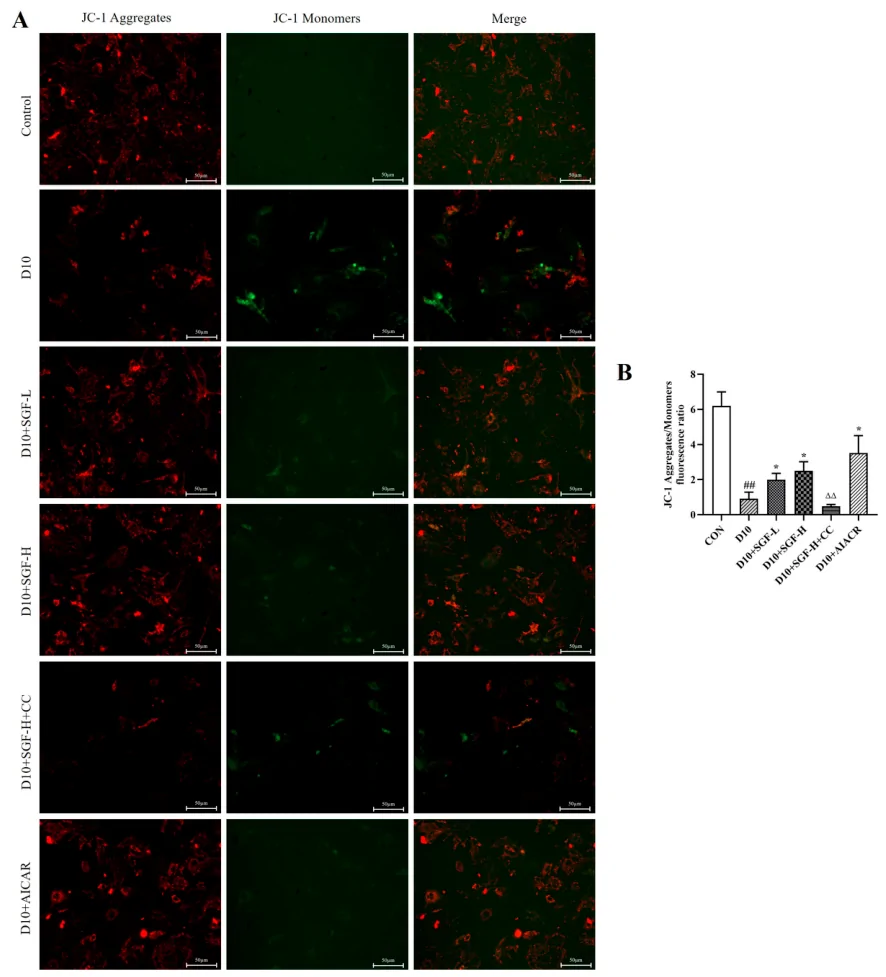

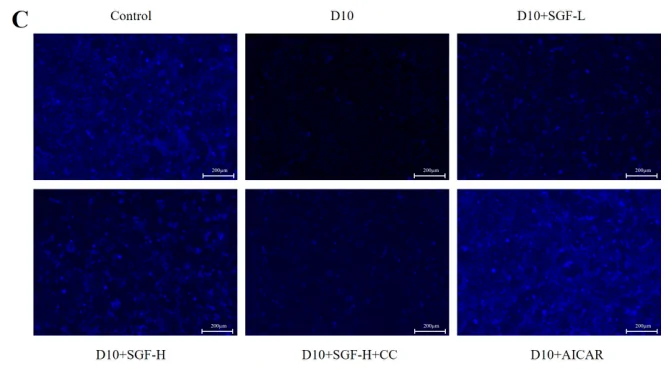

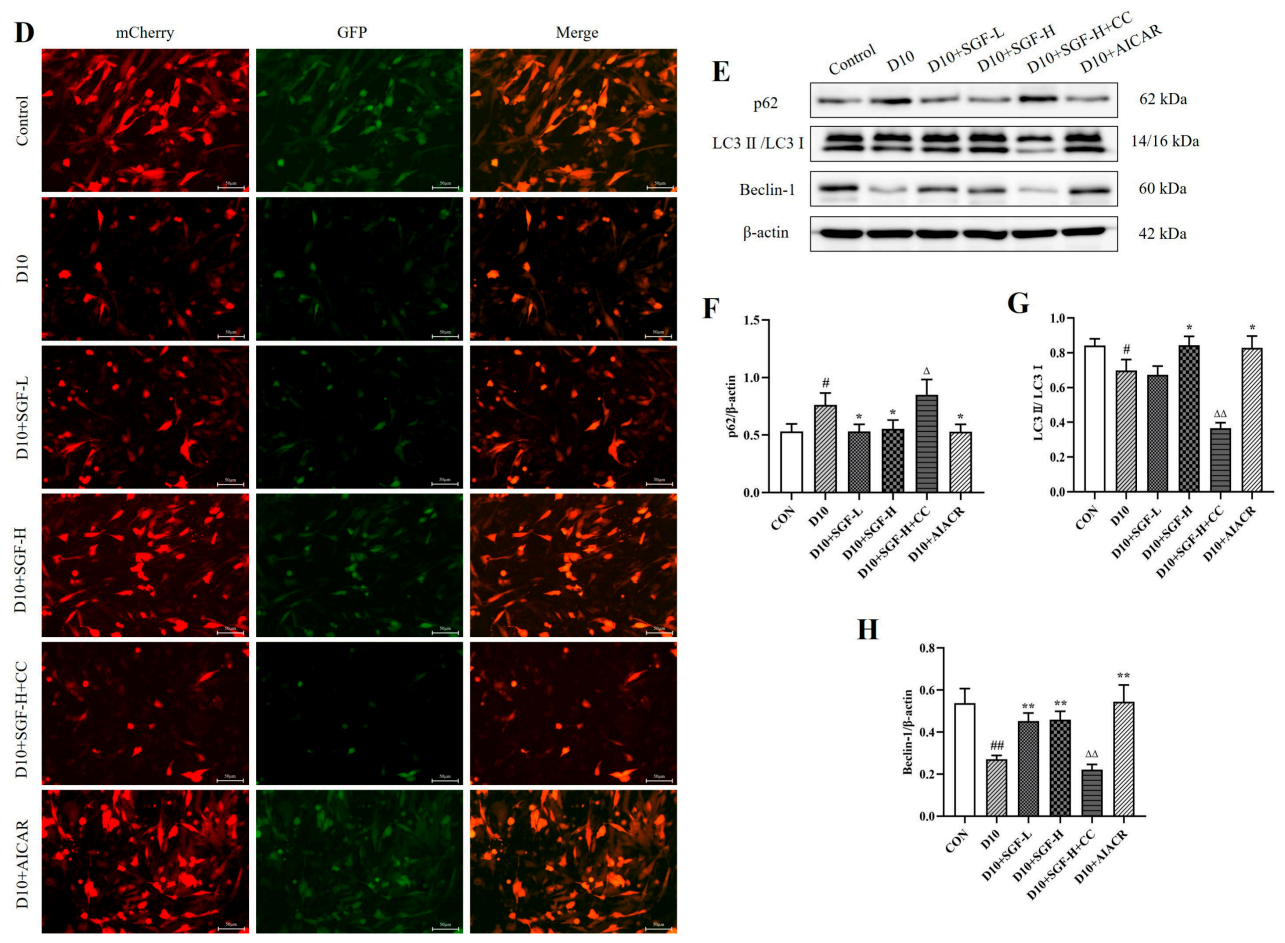

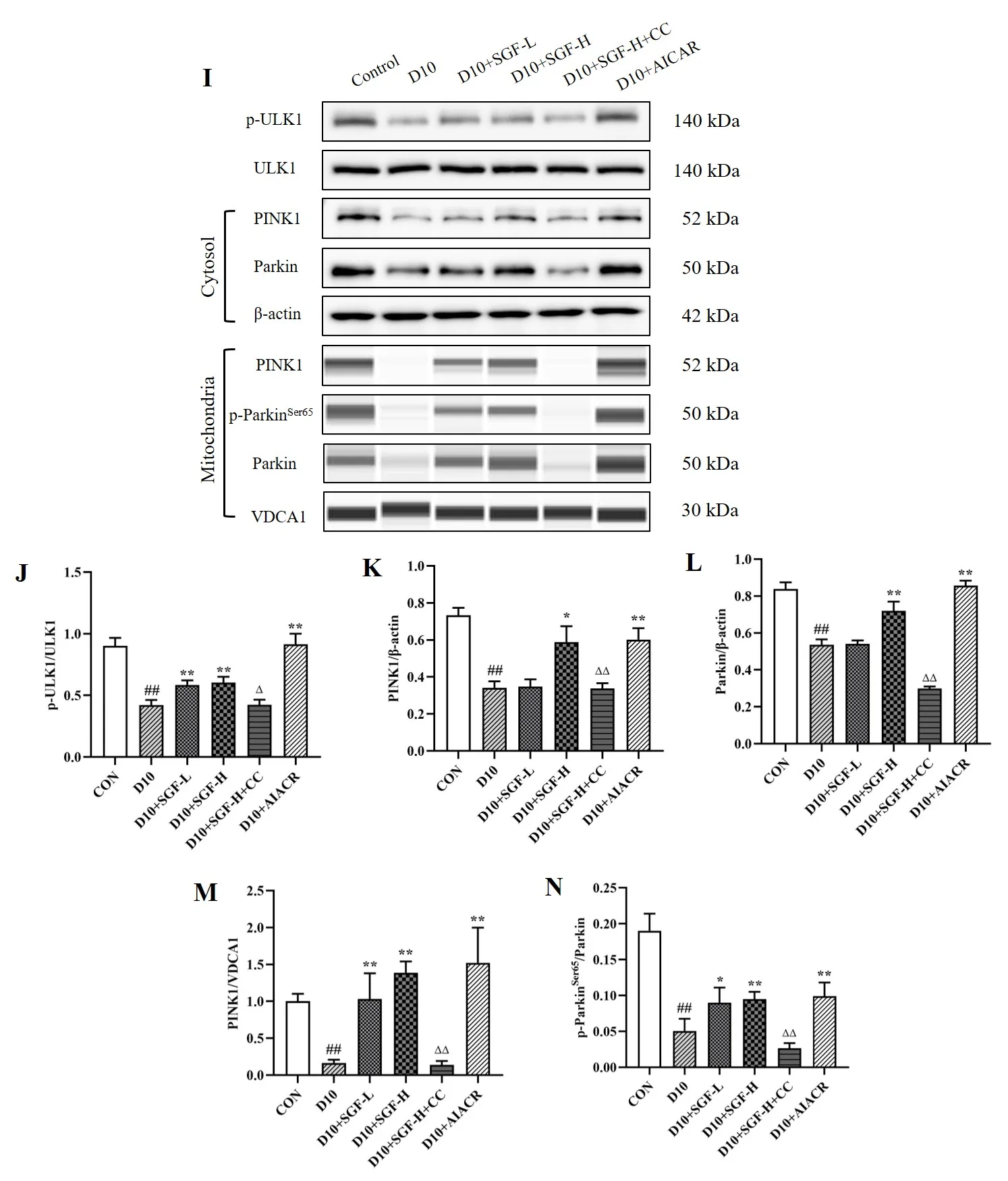

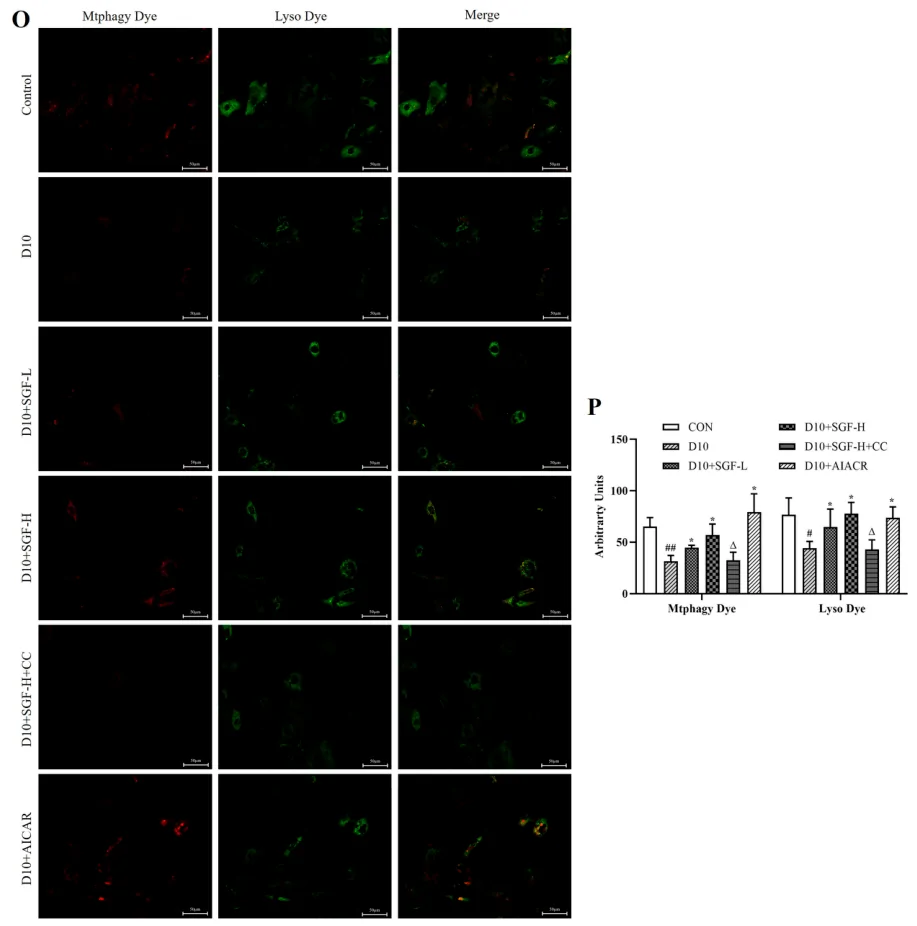
SGF upregulated the expression of key factors in the PINK1/Parkin signaling pathway in D-galactose-induced H9c2 cells in association with AMPK/ULK1 signaling. (**A**,**B**) JC-1 red/green fluorescence ratio in H9C2 cells (scale bars = 50 μm). (**C**) Representative images of MDC staining for autophagy (scale bars = 200 μm). (**D**) Fluorescence images after infection with Ad-mCherry-GFP-LC3B adenovirus for detecting cellular autophagy (scale bars = 50 μm). (**E**–**H**) Representative protein band images and quantitative analysis of the expression of autophagy-related proteins p62, LC3II/LC3I and Beclin-1. (**I**–**N**) Representative protein band images and quantitative analysis of p-ULK1/ULK1, and proteins related to the PINK1/Parkin-dependent mitophagy pathway. (**O**) Representative images of H9c2 cells stained with Mtphagy Dye and Lyso Dye following a separate CCCP challenge (scale bars = 50 μm). (**P**) Quantitative analysis of Mtphagy Dye and Lyso Dye in H9c2 cells. *n* = 3 for each group. # *p* < 0.05, ## *p* < 0.01 vs. control group, * *p* < 0.05, ** *p* < 0.01 vs. D10 group, ^∆^ *p* < 0.05, ^∆∆^ *p* < 0.01 vs. D10+SGF-H group.

**Figure 10:**
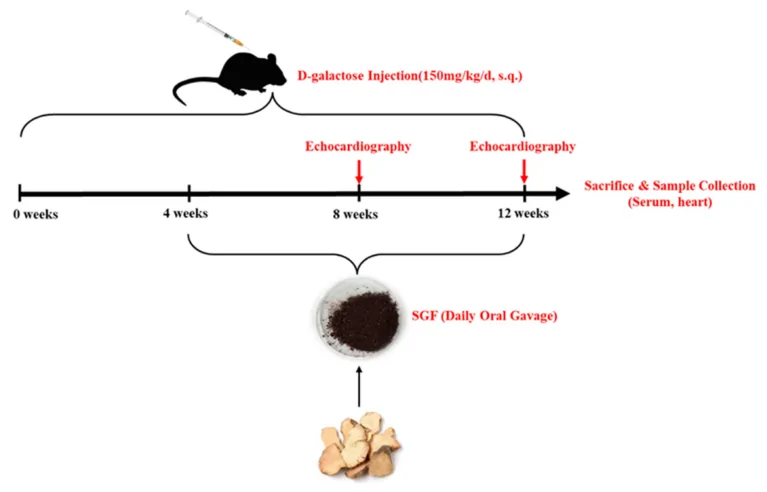
Intervention protocol and experimental flow chart in mice.

**Table 1:** Echocardiographic parameters of mice following 4 weeks of SGF treatment.

Groups	Control	D-gal	SGF-L	SGF-H
HR(bpm)	471.00 ± 28.00	468.38 ± 56.23	470.00 ± 36.26	470.25 ± 50.30
IVSd/cm	0.09 ± 0.02	0.12 ± 0.01 **^##^**	0.09 ± 0.01 **	0.08 ± 0.01 **
IVSs/cm	0.12 ± 0.02	0.15 ± 0.01 **^##^**	0.12 ± 0.01 **	0.12 ± 0.01 **
LVIDd/cm	0.37 ± 0.04	0.36 ± 0.05	0.35 ± 0.01	0.35 ± 0.01
LVIDs/cm	0.28 ± 0.04	0.26 ± 0.04	0.27 ± 0.02	0.26 ± 0.03
LVPWd/cm	0.07 ± 0.01	0.11 ± 0.01 **^##^**	0.08 ± 0.01 **	0.08 ± 0.01 **
LVPWs/cm	0.09 ± 0.02	0.13 ± 0.01 **^##^**	0.11 ± 0.01 **	0.11 ± 0.01 **
FS/%	22.62 ± 3.76	27.41 ± 5.04 **^#^**	24.26 ± 4.31	25.57 ± 5.73
LVEDV/mL	0.13 ± 0.04	0.12 ± 0.04	0.11 ± 0.01	0.11 ± 0.01
LVESV/mL	0.06 ± 0.03	0.05 ± 0.02	0.05 ± 0.01	0.05 ± 0.01
EF/%	51.98 ± 6.75	60.51 ± 6.87 **^#^**	54.82 ± 7.61	57.26 ± 9.03

Left ventricular end-diastolic interventricular septal thickness (IVSd), left ventricular end diastolic diameter (LVIDd), left ventricular end-diastolic posterior wall thickness (LVPWd), left ventricular end-systolic interventricular septal thickness (IVSs), left ventricular end-systolic diameter (LVIDs), and left ventricular end-systolic posterior wall thickness (LVPWs) were measured. The ejection fraction (EF), fraction shortening (FS), left ventricular end-diastolic volume (LVEDV), and left ventricular end-systolic volume (LVESV). # *p* < 0.05, ## *p* < 0.01 vs. control group, ** *p* < 0.01 vs. D-galactose group, *n* = 8 for each group.

**Table 2:** Echocardiographic parameters of mice following 8 weeks of SGF treatment.

Groups	Control	D-gal	SGF-L	SGF-H
HR(bpm)	484.38 ± 24.22	456.25 ± 48.05	441.13 ± 42.12	457.13 ± 37.36
IVSd/cm	0.08 ± 0.01	0.12 ± 0.01 **^##^**	0.08 ± 0.01 **	0.08 ± 0.01 **
IVSs/cm	0.12 ± 0.01	0.15 ± 0.01 **^##^**	0.11 ± 0.01 **	0.12 ± 0.02 **
LVIDd/cm	0.39 ± 0.03	0.30 ± 0.02 **^##^**	0.35 ± 0.04 *	0.36 ± 0.03 **
LVIDs/cm	0.30 ± 0.03	0.22 ± 0.02 **^##^**	0.26 ± 0.03 **	0.27 ± 0.03 **
LVPWd/cm	0.08 ± 0.01	0.12 ± 0.02 **^##^**	0.09 ± 0.01 **	0.09 ± 0.01 **
LVPWs/cm	0.10 ± 0.01	0.14 ± 0.01 **^##^**	0.11 ± 0.02 **	0.12 ± 0.02 *
FS/%	22.12 ± 0.93	27.07 ± 3.96 **^##^**	23.66 ± 2.15 *	24.23 ± 2.71
LVEDV/mL	0.15 ± 0.04	0.07 ± 0.01 **^##^**	0.11 ± 0.03 **	0.12 ± 0.03 **
LVESV/mL	0.07 ± 0.02	0.03 ± 0.01 **^##^**	0.05 ± 0.02 **	0.05 ± 0.02 **
EF/%	51.25 ± 1.78	60.08 ± 6.10 **^##^**	54.05 ± 3.80 *	53.09 ± 2.87 **

Left ventricular end-diastolic interventricular septal thickness (IVSd), left ventricular end diastolic diameter (LVIDd), left ventricular end-diastolic posterior wall thickness (LVPWd), left ventricular end-systolic interventricular septal thickness (IVSs), left ventricular end-systolic diameter (LVIDs), and left ventricular end-systolic posterior wall thickness (LVPWs) were measured. The ejection fraction (EF), fraction shortening (FS), left ventricular end-diastolic volume (LVEDV), and left ventricular end-systolic volume (LVESV). ## *p* < 0.01 vs. control group, * *p* < 0.05, ** *p* < 0.01 vs. D-galactose group, *n* = 8 for each group.

## Data Availability

The original contributions presented in this study are included in the article/Supplementary Materials. Further inquiries can be directed to the corresponding author.

## Supplementary Materials

The following supporting information can be downloaded at https://www.mdpi.com/article/10.3390/ijms27219969/s1.
