# Supplementary material for: Smilax glabra Roxb. Flavonoids Attenuate D-Galactose-Induced Cardiac Alterations and Are Associated with Modulation of the PGC-1α/NRF1/TFAM Axis and PINK1/Parkin Signaling Pathway in an AMPK-Associated Manner
Source: Int J Mol Sci. 2026 Nov 6;27(21):9969. doi: 10.3390/ijms27219969 (PMC13763908; doi:10.3390/ijms27219969)
Supplement: Supplementary file 1 [file ijms-27-09969-s001.zip › ijms-4614730-supplementary.pdf]

Article

Smilax glabra Roxb. flavonoids attenuate D-galactose-induced cardiac alterations and are associated with modulation of the PGC-1α/NRF1/TFAM axis and PINK1/Parkin signaling pathway in an AMPK-associated manner

Danting Fu, Jiajun Shi, Qinqin Yang, Chunwei Zheng and Yu Chen *

Department of Experimental Animals, Zhejiang Academy of Traditional Chinese Medicine, Hangzhou 310007, China; fdt1018@163.com (D.F.); xsrjsjj1992@126.com (J.S.); qqy1029@126.com (Q.Y.); zhengcw1972@163.com (C.Z.)

* Correspondence: chenyu97012064@zuaa.zju.edu.cn

Supplementary Figures

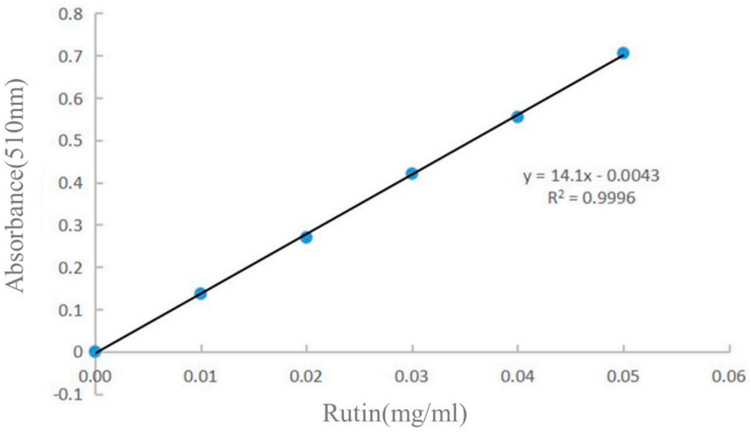

Figure S1. Standard graph of rutin. The total flavonoid content of SGF was measured using colorimetric method. A certain amount of rutin standard and SGF were precisely weighed, dissolved in 70% ethanol(v/v), and then a certain amount of the dissolved liquid was added to a 10ml volumetric flask. 70% ethanol(v/v) was added to make a volume of 5 mL. Firstly, 0.3 mL of 5% $NaNO_2$(w/v) was added to the volumetric flask, shaken well, and left to stand for 5 minutes. Then, 0.3 mL of 10% $Al(NO_3)_3$ (w/v) was added, shaken well, and left to stand for 6 minutes. After that, 2 mL of 4% NaOH(w/v) was added, and the solution was made up to the calibration mark with distilled water, shaken well, and left to stand for 10 minutes. Absorbance was read at 510 nm against the blank and the flavonoid content was calculated.

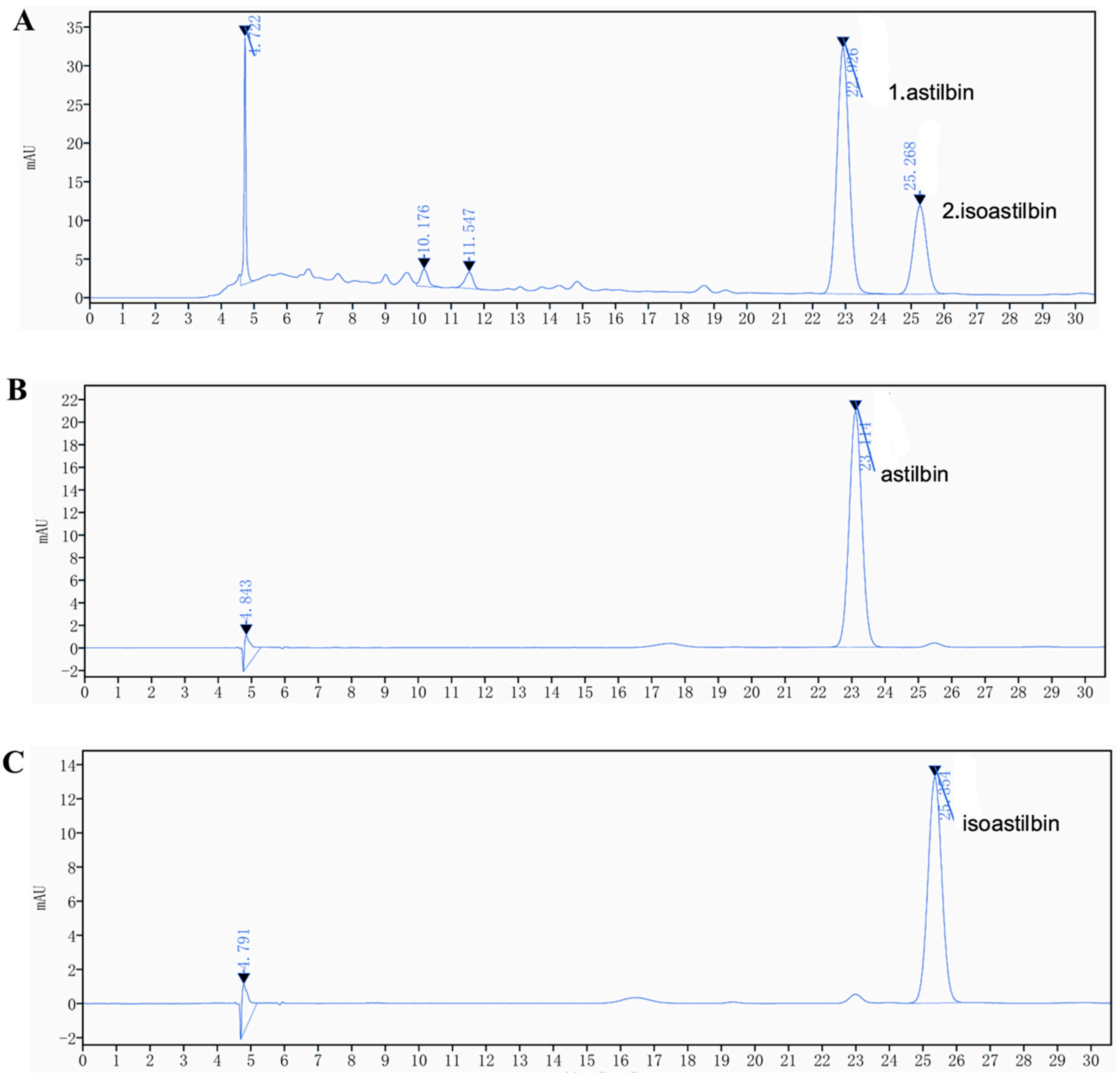

Figure S2. HPLC analysis of SGF. HPLC chromatograms of (A) SGF, (B) the astilbin standard compound and (C) the isoastilbin standard compound; According to the calculation, the percentage content of astilbin, isoastilbin in the dry powder of SGF was 18.92%, and 6.74%.
